# Gut microbiota dysbiosis affects intestinal sensitivity through epithelium-to-neuron signaling: novel insights from a colon organoid-based model to improve visceral pain therapy

**DOI:** 10.1080/19490976.2025.2547029

**Published:** 2025-09-03

**Authors:** Francesco Margiotta, Elena Lucarini, Alessandra Toti, Lorenzo Curti, Alessio Masi, Tommaso Mello, Gwenaelle Le Gall, Gianluca Mattei, Alberto Magi, David Vauzour, Guido Mannaioni, Lorenzo Di Cesare Mannelli, Carla Ghelardini

**Affiliations:** a Department of Neuroscience, Psychology, Drug Research and Child Health - NEUROFARBA - Pharmacology and Toxicology Section, University of Florence, Florence, Italy; b Department of Clinical and Experimental Biomedical Sciences “Mario Serio”, University of Florence, Florence, Italy; c Norwich Medical School, University of East Anglia, Norwich, UK; d Department of Health Sciences, Clinical Pharmacology and Oncology Unit, University of Florence, Florence, Italy; e Department of Information Engineering, University of Florence, Florence, Italy

**Keywords:** Visceral pain, organoids, epithelial-neuronal signaling, microbiota, dysbiosis, DRG neurons, intestinal epithelium

## Abstract

Chronic gastrointestinal pain is a hallmark of most intestinal pathologies, yet effective treatments remain elusive given the complexity of the underlying mechanisms. Aiming to investigate the intestinal epithelium contribution to visceral pain modulation in dysbiosis context, we first demonstrated that intracolonic instillation of microbe-free fecal supernatants from mice with post-inflammatory dysbiosis induced by dextran sodium sulfate (FS^DSS^) provokes visceral hypersensitivity in recipient mice. Epithelium involvement in the response to FS^DSS^ was analyzed through a novel *in vitro* approach comprising murine epithelial colon organoids and primary dorsal root ganglia (DRG) neurons. FS^DSS^ treatment induced growth and metabolic impairment in colon organoids, which revealed a dysbiosis-driven epithelial dysfunction. Notably, the combination of FS^DSS^ and conditioned medium from FS^DSS^-treated colon organoids induced an increase in DRG neuron intrinsic excitability, along with greater immunoreactivity to c-Fos and calcitonin-gene related peptide, implicating an integrated role of both microbial and epithelial products in visceral sensitivity regulation. By investigating the underlying signaling, metabolomic analysis revealed reduced levels of short chain fatty acids in FS^DSS^, such as butyrate, acetate, valerate, and propionate. Moreover, transcriptomic analysis of FS^DSS^-treated colon organoids showed the dysregulated expression of several signaling factors by which intestinal epithelium may modulate sensory neuron excitability, including proteases, cytokines, neuromodulators, growth factors, and hormones. These findings provide novel insights into the role of gut epithelium in the modulation of sensory neuron excitability under dysbiosis conditions, emphasizing that targeting epithelial-neuronal signaling might represent a promising therapeutic strategy for visceral pain management.

## 1. Introduction

Visceral pain, as reported in irritable bowel syndrome (IBS) or inflammatory bowel diseases (IBD), is one of the most common reasons for patients with gastrointestinal disorders to seek medical advice.^1–3^ The plethora of actors involved in the regulation of visceral sensitivity, including gut microbiome, intestinal epithelium, immune system, and nervous system, makes the treatment of abdominal pain challenging,^4–8^ and explain the current absence of fully effective therapies.^2^ Pain signaling within the colon mainly arises from visceral primary afferent neurons (i.e. nociceptors), which convey the information from the thoracolumbar and lumbosacral dorsal root ganglia (DRG) to the central ascending pain pathways through the spinal cord.^9,10^

Gut dysbiosis emerges as an important driver in the onset and persistence of visceral hypersensitivity.^5,11,12^ Indeed, microbial-derived products (i.e., short chain fatty acids (SCFAs), bile acids and amines) can directly influence the activity of chemosensitive nociceptors,^13–16^ especially in the “leaky gut” conditions associated with several gastrointestinal diseases.^17–19^ Concurrently, the epithelium has been reported to mediate the effect of microbiota-derived factors on intestinal afferents.^7,20–22^ Epithelial functions are strongly influenced by microbiota composition and metabolism as evidenced by the expressions of many receptors that can sense the luminal content and drive the signaling to different intestinal partners, including afferent neurons.^7,23,24^ Noteworthy, the use of optogenetic approaches demonstrated the active role of gut epithelium in modulating visceral sensitivity,^25,26^ providing the rationale for thinking of the epithelium as a therapeutic target. In a pathological scenario, nociceptor stimulation by luminal- and epithelium-derived compounds results in neuronal sensitization, increased neurotransmission to spinal cord and neurogenic inflammation.^6,27^ In fact, the release of certain neurotransmitters, such as calcitonin gene-related peptide (CGRP), from sensory neurons can amplify pain transmission as well as boost the peripheral inflammatory response.^4,28,29^

However, if and how dysbiosis of gut microbiota can affect the epithelium-to-neuron signaling involved in visceral sensitivity regulation still needs to be elucidated. Indeed, although *in vivo* studies have reported the importance of intestinal epithelium in visceral pain, the crosstalk between epithelium and sensory neurons remains difficult to study due to the interference of numerous variables which do not permit the dissection of molecular signals among the different cell types inhabiting the gut.^30^

Starting from the *in vivo* demonstration that the intracolonic instillation of microbe-free fecal supernatants from mice with post-inflammatory dysbiosis induced by dextran sodium sulfate is enough to transfer visceral hypersensitivity into naïve recipient mice, our work aimed to assess the changes in colon epithelium metabolism and signaling determined by the dysbiosis condition and their impact on the intrinsic excitability of sensory neurons. This objective has been achieved by using an innovative *in vitro* approach, combining murine epithelial colon organoids and primary DRG neurons. Finally, our investigations revealed pharmacologically modulable mechanisms by which the colonic epithelium can influence neuronal sensitivity, offering novel targets for the development of new therapeutic strategies against gastrointestinal pain.

## 2. Materials and methods

### 2.1. Animals

Male C57BL/6N mice aged 6–8 weeks were purchased from Charles River Laboratories (Lecco, Italy). Animals were housed in CeSAL (Centro Stabulazione Animali da Laboratorio, University of Florence), kept at 23 ± 1°C with a 12 h light/dark cycle, light at 7 a.m., and were fed a standard laboratory diet (Teklad Global Diet; 18.5% proteins, 5.5% fat, #2018, Inotiv produced from Mucedola, Milan, Italy) and tap water *ad libitum*. All animal experiments were conducted in compliance with the Directive 2010/63/EU of the European parliament and of the European Union council (22 September 2010) on the protection of animals used for scientific purposes, as well as in accordance with the guidelines of the International Association for the Study of Pain (IASP). The ethical policy of the University of Florence complies with the Guide for the Care and Use of Laboratory Animals of the US National Institutes of Health (NIH Publication No. 85–23, revised 1996; University of Florence assurance number: A5278-01). The experiments received formal approval from the Italian Ministry of Health (No. 17E9C.N.B5Z and 1046/2023-PR) and from the Animal Subjects Review Board of the University of Florence. Animal experiments have been reported in accordance with the ARRIVE 2.0 guidelines.^31^ Every effort was made to minimize animal suffering and to reduce the number of animals used in the study.

### 2.2. Dextran sodium sulfate (DSS)-induced colitis and dysbiosis model

Experimental colitis and associated dysbiosis were induced following previously described methods with slight modifications.^32,33^ Briefly, mice were given 2.5% (w:v) dextran sodium sulfate (DSS) (AbMole BioScience, Houston, TX, USA) in tap water *ad libitum* for five days, followed by DSS-free tap water in the next three days. Control mice received tap water *ad libitum* for the duration of the experiment. All experiments were performed using the same lot of DSS (M9443-202416-1). Disease severity of fecal supernatant donors was monitored by percent body weight change and Disease Activity Index (DAI; assessing stool consistency, occult/gross bleeding, and weight loss) as described by Simeoli et al.^32^ with minor modifications. Body weight loss was scored as follows: 0 = no loss; 1 = 1–3%; 2 = 3–6%; 3 = 6–9%; 4 =  > 9%. Stool consistency was evaluated on a scale of 0 to 4: 0 = normal; 2 = loose stool; 4 = diarrhea. Fecal blood was assessed using the following scale: 0 = none; 2 = visible blood; 4 = gross bleeding (fresh perianal blood). The total DAI score, with a maximum value of 12, was used as an indicator of intestinal inflammatory activity (Figure S1).

### 2.3. Preparation of fecal supernatants (FS) for *in vivo* studies and intracolonic injection in mice

Fecal samples were collected from control and DSS-treated mice two and three days after the end of the DSS treatment, pooled and frozen at −80°C. On the day of injection, feces were homogenized in saline solution (100 mg/mL) and centrifuged at 700×g for 2 minutes. Fecal supernatants (FS) were collected and filtered through a 0.22 µm filter unit, to obtain microbe-free FS from healthy (FS^CTR^) and DSS-treated mice (FS^DSS^). Fresh FS (300 µL) were slowly intracolonically infused for 1 minute in mice under anesthesia (2% isoflurane) by using a flexible gavage needle (Instech Laboratories, Plymouth Meeting, PA, USA) inserted at 1 cm from the anus. Following completion of the procedure, animals were held inclined with their heads downwards for 1 minute in order to prevent fluid leakage from the rectum. FS injection in mice was performed once daily for 4 consecutive days. Control animals were infused with saline solution.

### 2.4. Assessment of visceral sensitivity by abdominal withdrawal reflex (AWR)

Behavioral responses to colorectal distension (CRD) were evaluated by measuring the abdominal withdrawal reflex (AWR) using a semiquantitative scoring system in conscious animals.^34^ Mice were anesthetized with 2% isoflurane (VIRBAC S.r.l., Milan, Italy) and a lubricated latex balloon connected to polyethylene tubing, assembled into an embolectomy catheter (Fogarty 4F; Edwards Lifesciences, Milan, Italy) and a water-filled syringe was inserted via the anus into the rectum and descending colon. The tubing was secured to the tail to maintain balloon placement. Following a 30-minute recovery period, abdominal withdrawal reflex (AWR) was assessed in conscious animals in response to graded CRD (50, 100, 150, and 200 µL). Blinded observers assigned AWR scores based on behavioral responses: no response (0); immobility with occasional head clenching at stimulus onset (1); mild abdominal muscle contraction without abdominal lifting (2); strong contraction with abdominal lifting (3); body arching with elevation of pelvic structures and scrotum (4). A 3-minute interval was maintained between consecutive distensions.

### 2.5. Assessment of visceral sensitivity by visceromotor response (VMR)

Visceromotor response (VMR) to CRD was employed as an objective measure of visceral sensitivity as previously described.^34^ Briefly, two electromyographic (EMG) electrodes were surgically implanted into the external oblique abdominal muscle under 2% isoflurane anesthesia (VIRBAC S.r.l., Milan, Italy) and exteriorized dorsally one week prior to testing. On the day of the experiment, a lubricated latex balloon connected to polyethylene tubing, assembled into an embolectomy catheter (Fogarty 4F; Edwards Lifesciences, Milan, Italy) and a water-filled syringe, was inserted via the anus into the rectum and descending colon of animals under 2% isoflurane anesthesia. The tubing was secured to the tail to maintain the balloon’s position. After a 30-minute recovery period, CRD was performed filling the syringe with graded water volumes (50, 100, 150, and 200 µL). EMG signals were acquired via a data acquisition system, amplified, filtered (Animal Bio Amp, ADInstruments, Oxford, UK), digitized (PowerLab 4/35, ADInstruments), and analyzed using LabChart 8 (ADInstruments). VMR magnitude was quantified by calculating the area under the curve (AUC) of the EMG signal during distension (30 s), subtracting the AUC from the baseline period (30 s), and expressing the result as a percentage increase from baseline. A 3-minute interval was maintained between successive distensions.

### 2.6. Murine colon organoid culture protocol

Murine colon organoids were obtained following the method described by Fan et al. with some adaptations.^35^ Briefly, the colon was removed from mice and washed with ice cold PBS supplemented with 100 U/mL penicillin and 100 µg/mL streptomycin (Merck, Milan, Italy). Crypts were isolated by a non-enzymatic reaction (20 mM EDTA in PBS for 35 minutes at 37°C) followed by vortexing to generate four different fractions. Crypts were then embedded in Matrigel® Growth Factor Reduced Basement Membrane Matrix (356231, Corning, Tewksbury, MA, USA) supplemented with 50 ng/mL Recombinant Murine EGF (Peprotech-Life Technologies, Milan, Italy), 500 ng/mL Recombinant Human R-Spondin-1 (Peprotech-Life Technologies, Milan, Italy), 100 ng/mL Recombinant Murine Noggin (Peprotech-Life Technologies, Milan, Italy), 100 ng/mL Recombinant Murine Wnt-3a (Peprotech-Life Technologies, Milan, Italy), *N*-2 Supplement (Gibco-Life Technologies, Milan, Italy), B-27 Supplement serum free (Gibco-Life Technologies, Milan, Italy) and 1 µM N-Acetyl-L-cysteine (Merck, Milan, Italy), and plated as 50 µL droplets in 24-well plates (Corning, Tewksbury, MA, USA) with a density of ~ 1000 crypts/well. After 30 minutes of Matrigel polymerization, organoid medium (Advanced DMEM/F-12 (ADF) (Gibco-Life Technologies, Milan, Italy) supplemented with 100 U/mL penicillin, 100 µg/mL streptomycin, 2 mM GlutaMax supplement (Gibco-Life Technologies, Milan, Italy), 10 µM HEPES, 50 ng/mL Recombinant Murine EGF, 500 ng/mL Recombinant Human R-Spondin-1, 50 ng/mL Recombinant Murine Noggin, 100 ng/mL Recombinant Murine Wnt-3A, *N*-2 Supplement, B-27 Supplement serum free and 1 µM N-Acetyl-L-cysteine) was added. The medium was changed every two-three days.

For passaging, Matrigel domes were disrupted with ice cold PBS, and organoids were collected in 15 mL tubes and centrifuged at 200×g, for 5 minutes at 4°C. This step was repeated twice. Isolated organoids were incubated with Cell Recovery solution (Corning, Tewksbury, MA, USA) for 30 minutes at 4°C to remove Matrigel. Then, ice cold PBS was added, and organoids were centrifuged twice at 100×g, for 5 minutes at 4°C. Where not specified, organoids were plated as 50 µL droplets in 24-well plates with a density of ~ 750 organoids/well. Organoids were maintained in humidified incubators at 37°C in 5% CO_2_. Experimental analysis on colon organoids were always performed after one passage step.

### 2.7. Primary murine dorsal root ganglia (DRG) neuron culture protocol

Primary murine dorsal root ganglia (DRG) neurons were obtained as described by Perner and Sokol with some adaptations.^36^ Briefly, the spinal column was removed from mice and cut frontally in half. All DRGs were collected in ice cold Dulbecco’s Modified Eagle Medium (DMEM) – high glucose (Merck, Milan, Italy), supplemented with 10% Fetal Bovine Serum (FBS) (Euroclone, Milan, Italy), 100 U/mL penicillin, 100 µg/mL streptomycin, and 2 mM L-Glutamine and then digested in an enzymatic solution consisting of 1.25 mg/mL Collagenase A (Merck, Milan, Italy) and 2.5 mg/mL Dispase II (Merck, Milan, Italy) for 30 minutes in the cell incubator. After the digestion, DRGs were triturated 10–20 times through three different needles (18, 23, and 26 gauge). Where not specified, around 2.5 × 10^3^ DRG neurons were plated on coverslips (Ø 13 mm) previously coated with 30 µL laminin (10 µg/mL) (Merck, Milan, Italy) in 24-well plate and cultured in Neurobasal-A medium (Gibco-Life Technologies, Milan, Italy) supplemented with 100 U/mL penicillin, 100 µg/mL streptomycin, 2 mM GlutaMax supplement, B-27 supplement serum free and 50 ng/mL Recombinant Murine β-NGF (Invitrogen-Life Technologies, Milan, Italy).

### 2.8. Preparation of fecal supernatants (FS) for *in vitro* studies and treatment

Fecal pellets were collected two and three days after the end of DSS treatment from healthy and DSS-treated mice, pooled and frozen at −80°C. Feces were homogenized in ADF supplemented with 100 U/mL penicillin, 100 µg/mL streptomycin, 2 mM GlutaMax supplement and 10 µM HEPES at 200 mg/mL and centrifuged at 700×g for 2 minutes. Fecal supernatants (FS) were collected and filtered through a 0.22 µm filter unit, to obtain microbe-free FS from healthy (FS^CTR^) and DSS-treated mice (FS^DSS^). 2% fresh FS (4 mg/mL) were immediately used to treat colon organoids or DRG neurons. For colon organoids, FS were added to freshly renewed organoid medium on days 3 and 5 from the seeding (day 1). For DRG neurons, FS treatments were fully done in organoid medium or undiluted conditioned medium from organoids for 48 hours, starting 24 hours after the isolation (see “2.9 Preparation of conditioned media (CM) from organoids and treatment”).

### 2.9. Preparation of conditioned media (CM) from organoids and treatment

After five days of treatment with FS^CTR^ and FS^DSS^, the organoid medium was totally replaced with fresh organoid medium. After 24 hours of conditioning, conditioned media (CM) from FS^CTR^-treated organoids (CM^FS CTR^) and FS^DSS^-treated organoids (CM^FS DSS^) were collected, centrifuged at 200×g for 5 minutes and transferred to new tubes. Undiluted CM were immediately used alone or supplemented with 4 mg/mL FS to treat DRG neurons for 48 hours, 24 hours after their isolation.

### 2.10. Colon organoid immunofluorescence and confocal imaging

~50 colon organoids/well were plated on μ-slide 8 well chambers (IBIDI, Gräfelfing, Germany) coated with 3 mg/mL of Matrigel and organoid medium was added. After overnight incubation, the medium was removed, organoids were washed with PBS and fixed with 4% paraformaldehyde for 10 minutes at room temperature. Fixed organoids were washed three times with PBS for 5 minutes before incubating them with the permeabilization solution (0.2% Triton X-100 in PBS) for 1 hour at room temperature. After permeabilization, organoids were incubated in blocking solution (5% bovine serum albumin (BSA) + 0.01% Triton X-100 in PBS) for 3 hours. Organoids were subsequently incubated with the following primary antibodies: 1:200 Goat anti-Mouse E-Cadherin (AF748, Lot CYG0420091, R&D Systems-Bio-Techne, Milan, Italy); 1:200 Rabbit anti-Mouse Mucin 2 (GTX100664, Lot 44,447, GeneTex, Irvine, CA, USA); 1:100 Rabbit anti-Mouse Carbonic Anhydrase IV (PA5-81329, Lot WA3152375A, Invitrogen-Life Technologies, Milan, Italy); 1:200 Rabbit anti-Mouse Chromogranin A (NB120-15160, Lot C-2, Novus Biologicals-Bio-Techne, Milan, Italy), and 1:200 Rabbit anti-Mouse LGR5 (GTX130204, Lot 42,060, GeneTex, Irvine, CA, USA) in blocking solution overnight at 4°C. The following day, organoids were washed three times with PBS for 5 minutes and then they were incubated in the dark with the following secondary antibody: 1:500 Donkey anti-Goat IgG Alexa Fluor™ 568 (A-11057, Invitrogen-Life Technologies, Milan, Italy) and 1:500 Goat anti-Rabbit IgG Alexa Fluor™ 488 (A-11034, Invitrogen-Life Technologies, Milan, Italy) in blocking solution for 2 hours at room temperature. After three PBS washes for 5 minutes, organoids were incubated with DAPI, a nuclei-marker, for 5 minutes at room temperature. Negative control samples (no exposure to the primary antisera) were processed concurrently with the other organoids for all immunohistochemical studies. The images were acquired using the confocal microscope Leica SP8 AOBS equipped with a supercontinuum white light laser (WLL) (Leica Microsystems, Mannheim, Germany) through a 20× objective.

### 2.11. Colon organoid and DRG neuron viability assay

Cell viability was evaluated by the reduction of 3-(4,5-dimethylthiozol-2-yl)-2,5-diphenyltetrazolium bromide (MTT) (Merck, Milan, Italy).

Colon organoids were plated as 7 µL droplets at a density of ~ 100 organoids/well in 96-well plates. At the end of the treatment, colon organoids were incubated for 3 hours in the cell incubator with 1 mg/mL MTT in phenol red-free DMEM (Merck, Milan, Italy). Matrigel domes were dissolved by incubating organoids with 2% sodium dodecyl sulfate (SDS) (Merck, Milan, Italy) for 2 hours in the cell incubator and then the colored formazan crystals were dissolved with dimethyl sulfoxide (DMSO) (Merck, Milan, Italy) for 1 hour. Absorbance was measured at 570 nm and normalized on the numbers of organoids counted subsequently in each well. Values were measured in at least six wells for each condition in three experimental replicates.

Around 2 × 10^3^ DRG neurons were plated in laminin-coated 96-well plate and cultured in Neurobasal-A medium supplemented with 100 U/mL penicillin, 100 µg/mL streptomycin, 2 mM GlutaMax supplement, B-27 supplement serum free and 50 ng/mL Recombinant Murine β-NGF. At the end of the treatment, DRG neurons were incubated for 45 minutes in the cell incubator with 1 mg/mL MTT in phenol red-free DMEM/F12 (Gibco-Life Technologies, Milan, Italy). The colored formazan crystals were dissolved with DMSO for 10 minutes on an orbital shaker. Absorbance was measured at 570 nm. Values were measured in three-five technical replicates.

### 2.12. Morphometric analysis of colon organoids

The bright-field images of organoids were taken using an optical microscope equipped with a Nikon D5000 camera (Nikon, Amstelveen, The Netherlands). Five fields of view at 20× containing one organoid for each well, and an average of six wells were analyzed for each condition in five replicates. ImageJ software (Nation Institutes of Health, USA) was used to measure the growth area of each organoid (in µm^2^).

### 2.13. Electrophysiological recordings on DRG neurons

Whole-cell electrophysiological recordings were performed on DRG neurons, cultured on Ø 13 mm coverslips, previously exposed to FS and/or CM for 48 hours. Single coverslips were transferred to a flow chamber perfused with an extracellular solution composed of (in mM): NaCl (140), CaCl2 (2), MgCl2 (1), KCl (3), HEPES (10), D-(+) glucose (10) (pH 7.3–7.4 with NaOH). The flow chamber was positioned under the objective of an upright microscope (Nikon Eclipse E600FN, Nikon, Amstelveen, The Netherlands) equipped with infrared digital imaging. Patch-clamp pipettes were made from thin-walled borosilicate capillaries (Harvard Apparatus, Holliston, MA, USA) using a vertical puller (Narishige PP830) (Narishige International Limited, London, UK) and back-filled with an intracellular solution composed of (in mM): K+ gluconate (120), KCl (15), HEPES (10), EGTA (1), MgCl2 (2), Na2 phosphocreatine (5), NaGTP (0.3), MgATP (4) (pH 7.3–7.4), resulting in a bath resistance of 3–5 MΩ. Electrical signals were sampled at 10 kHz and low-pass filtered at 3 kHz with an AxonMulticlamp 700B (Molecular Devices, Sunnyvale, CA, USA). DRG neurons were characterized for each experimental condition by measuring passive properties such as membrane resistance, membrane capacitance and resting membrane potential. Intrinsic excitability was studied by measuring the number and threshold of action potentials generated in response to square current clamp pulses of increasing amplitude (25 pA, 1 s step). Six-twelve neurons were analyzed for each condition in four experimental replicates.

### 2.14. DRG neuron immunofluorescence and imaging

At the end of the treatment, DRG neurons were washed with PBS and fixed with 4% paraformaldehyde for 15 minutes at room temperature. Fixed neurons were washed three times with PBS for 5 minutes before incubating them with the permeabilization solution (0.3% Triton X-100 in PBS) for 10 minutes at room temperature. After permeabilization, DRG neurons were incubated in blocking solution (0.5% BSA + 0.3% Triton X-100 in PBS) for 30 minutes. Then, cells were incubated with the following primary antibodies: 1:100 Rabbit anti-Mouse c-Fos (BS-0469 R, Lot AI08094815, Bioss Antibodies, Woburn, MA, USA), 1:500 Goat anti-Mouse CGRP (PA1-85250, Lot XL3781521A, Invitrogen-Life Technologies, Milan, Italy) and 1:100 biotin-conjugated isolectin B4 (IB4) from *Griffonia simplicifolia* (I21414, Lot 2,349,068, Invitrogen-Life Technologies, Milan, Italy) in blocking solution overnight at 4°C. The following day, DRG neurons were washed three times with PBS for 5 minutes and then they were incubated in the dark with 1:500 Donkey anti-Goat IgG Alexa Fluor™ 568 (A-11057, Invitrogen-Life Technologies, Milan, Italy) in blocking solution for 2 hours at room temperature. After 3 washing steps with PBS for 5 minutes, the slides were incubated in the dark for further 2 hours at room temperature with 1:500 Goat anti-Rabbit IgG Alexa Fluor™ 488 (A-11034, Invitrogen-Life Technologies, Milan, Italy) and 1:500 Streptavidin Alexa Fluor™ 647 conjugate (S21374, Invitrogen-Life Technologies, Milan, Italy) in blocking solution. After three PBS washes for 5 minutes, the slides were mounted using Fluoroshield™ with DAPI (Merck, Milan, Italy). Negative control samples (no exposure to the primary antisera) were processed concurrently with the other neurons for all immunohistochemical studies. Images were acquired using a motorized Leica DM6000 B microscope equipped with a DFC350FX camera (Leica Microsystems, Mannheim, Germany). Two-five fields of view at 20× for each slide, and an average of 4–6 different slides were used for the quantitative analysis of c-Fos and CGRP, while images at 40× were acquired for the illustrative panel.

Quantitative analysis of c-Fos and CGRP was performed using ImageJ software.^37^ Region of interests (ROI) manager was used to identify each neuron. The fluorescence intensity was measured for each ROI, and the background signal was removed for each channel. The mean of the ROI fluorescence intensities was calculated for each slide and the results are reported as the mean of the mean of 4–6 different slides for each condition.

Subpopulation analysis was performed by classifying DRG neurons in four subpopulations according to the CGRP expression or IB4 binding ability: peptidergic C fibers (CGRP^+^),^38^ non-peptidergic C fibers (IB4^+^),^39^ Aδ fibers (IB4^+^/CGRP^+^) and double negative fibers, that could represent the Aβ fibers (IB4^−^/CGRP^−^).^40^ For each marker, all neurons with a fluorescence intensity greater than the 25^th^ percentile of the fluorescence intensity distribution were regarded as positive to the staining. c-Fos fluorescence intensity was measured in stained cells of each subpopulation and reported as the median of the fluorescence intensity distribution.

### 2.15. RNA sequencing and bioinformatics

At the end of the treatments, colon organoids were harvested in 15 mL tubes after disrupting Matrigel domes with ice cold PBS. Organoids were centrifuged three times at 200×g, for 5 minutes at 4°C, and then they were incubated with Cell Recovery solution for 30 minutes at 4°C to remove Matrigel. Then, ice cold PBS was added again, and organoids were centrifuged two times at 200×g, for 5 minutes at 4°C. Organoid pellets were immediately frozen at −80°C and shipped to BGI Tech Solutions (Hong Kong, China) for RNA extraction, library construction and RNA sequencing. Four replicates for each condition were used for this analysis. Concentration and quality of the extracted RNA were analyzed through the Bioanalyzer 2100 System (Agilent Technologies, Santa Clara, CA, USA). Amplification and library construction were performed following the pipeline for DNBSEQ Eukaryotic Stranded Transcriptome Sequencing. Sequencing was performed using a DNBSEQ platform (DNBSEQ Technology), generating 30 million paired-end reads with 150 base pairs.

FastQC (0.11.5) was employed to assess the quality of RNA sequencing data. Raw counts for each condition were obtained using Salmon (1.10.1) with paired-end FASTQ files, utilizing the *Mus musculus* GRCm39 reference transcriptome. Raw counts were imported into R using the tximport package (1.24.0) and subsequently normalized using DESeq2 (1.36.0), following the standard pipeline. To identify differentially expressed genes (DEGs), results were filtered using a *p* value threshold of ≤0.05. Gene set enrichment analysis (GSEA) was performed using the Wald test statistics from unfiltered DESeq2 results. The GSEA tool was used to query the Hallmarks and Gene Ontology Biological Process (GO:BP) databases from the Mouse Molecular Signatures Database (MSigDB). GSEA results were filtered based on the adjusted *p* value, and the normalized enrichment scores were plotted. All statistical analyses were conducted using R, including the aforementioned packages and any additional packages relevant to the GSEA implementation.

### 2.16. ^1^H NMR metabolomics

FS and CM were prepared as described above (see “2.8 Preparation of fecal supernatants (FS) and treatment” and “2.9 Preparation of conditioned media (CM) from organoids and treatment”). Five replicates for each condition were used for this analysis. Metabolites were analyzed and quantified by ^1^H NMR analysis. The preparation method was as previously described.^41,42^ Conditioned media were diluted to a ratio of 13 by adding deuterated phosphate buffer (1.9 mM Na2HPO4, 8.1 mM NaH2PO4, and 1 mM sodium 3-(trimethysilyl)-propionate-d4 in deuterated water (Goss Scientifics, Crewe, United Kingdom)). Following mixing and centrifugation, 500 μl of the supernatant was transferred into a 5 mm NMR tube for spectral acquisition. High-resolution ^1^H NMR spectra were acquired on a 600 MHz Bruker Avance spectrometer equipped with a 5 mm TCI proton-optimized triple resonance NMR inverse cryoprobe and a 24-slot autosampler (Bruker, Rheinstetten, Germany). Samples were maintained at 300 K during acquisition. Each spectrum was recorded using 128 scans with 65,536 complex data points and a spectral width of 20 ppm (acquisition time: 2.6 s). The noesypr1d presaturation sequence was employed to suppress the residual water signal, using low-power selective irradiation at the water resonance frequency during the recycle delay (D1 = 2 s) and the mixing time (D8 = 0.01 s). A 90° pulse length of 11.4 μs was applied to all samples. Spectral processing included a 0.1 Hz line broadening, zero filling, manual phasing, baseline correction, and referencing to the methyl signal of trimethylsilylpropanoic acid (TSP) at 0 ppm. Metabolite identification was based on literature and database references (Human Metabolome Database, https://www.hmdb.ca/), and quantification was performed using Chenomx® NMR Suite 8.6™.

### 2.17. Statistical analysis

The analysis of variance was performed by one-way ANOVA with Bonferroni’s significant difference procedure used for post-hoc comparisons. Results of DRG neuron subpopulation analysis are displayed as box and whiskers plot (median, percentiles and percentiles +1.5 interquantile range), with analysis of variance performed by Kruskal-Wallis test followed by Dunn’s test for post-hoc comparisons. *p* values < 0.05 were considered significant. Data were analyzed using OriginPro 10.1.5.132 software (OriginLab). Statistical analysis of metabolomics data was carried out using MetaboAnalystR Package. Data was normalized by median, log10 transformed and scaled by Pareto scaling (mean-centered and divided by the square root of the standard deviation of each variable). Results are presented as volcano plot combining results from both Fold Change (FC) and T-tests analyses. Partial Least-Squares Discriminant Analysis (PLS-DA) was employed to illustrate the clustering of different metabolites across groups. Adjusted *p* values of less than 0.1 were considered statistically significant.

## 3. Results

### 3.1. Fecal supernatants from DSS-treated mice induce visceral hypersensitivity in naïve recipient animals

**Figure 1.**
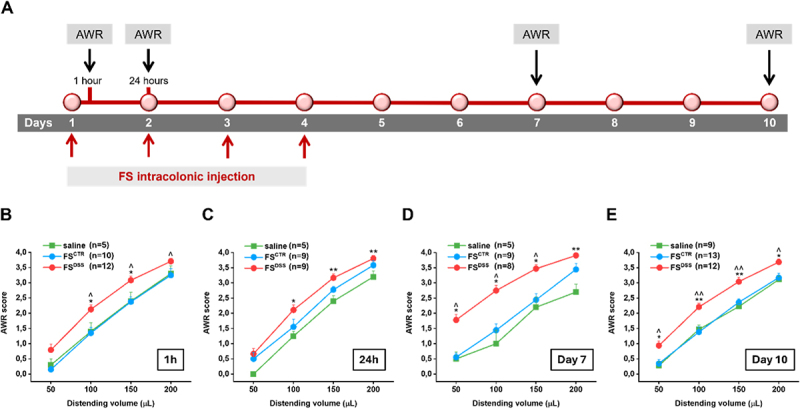
Effects of FS^CTR^ and FS^DSS^ intracolonic injection on visceral sensitivity of naïve mice - assessment of abdominal withdrawal reflex to colorectal distension. A) FS^CTR^ and FS^DSS^ (300 µL 100 mg/mL) were injected in naïve animals once daily for 4 consecutive days and AWR was assessed 1 and 24 hours after the first FS injection, 3 and 7 days after the last FS injection, as reported in the scheme. Visceral sensitivity was measured by evaluating the AWR (score 0–4) in response to colorectal distension (50–200 µL) B) 1 and C) 24 hours after the first FS injection, D) 3 and E) 7 days after the last FS injection. Values represent the mean ± SEM of each experimental group. The analysis of variance was performed by one-way ANOVA followed by Bonferroni post hoc comparison.

To investigate whether colon exposure to fecal products can directly affect visceral sensitivity, we intracolonically injected fecal supernatants from healthy (FS^CTR^) and DSS-treated mice (FS^DSS^) in naïve recipient mice once daily for 4 days. Visceral pain response was monitored in animals 1 and 24 hours after the first FS injection, 3 and 7 days after the last FS injection, by assigning a score to mice abdominal withdrawal reflex (AWR; 0–4) induced by colorectal distension (50–200 µL; Figure 1(A)). One hour after the FS intracolonic infusion, AWR elicited by the distension with 100–200 µL was significantly higher in the FS^DSS^ group than in saline and/or FS^CTR^ groups (Figure 1(B)). After 24 hours, AWR was still significantly increased in mice exposed to FS^DSS^ compared to those infused with saline but not compared to those infused with FS^CTR^ (Figure 1(C)). On day 7, 3 days after the last repeated treatment, visceral sensitivity in FS^DSS^ group appeared further increased, as AWR was significantly higher than in both control groups (saline and FS^CTR^), even for the smallest stimulus applied to the colon (50 µL; Figure 1(D)). The effect was maintained on day 10, 7 days after the last repeated treatment, although visceral sensitivity slightly decreased in the animals treated with FS^DSS^ (Figure 1(E)). The assessment of visceral sensitivity through the measure of visceromotor response (VMR) to colorectal distension in the same experimental conditions showed similar results. The entity of VMR was greater in the animals treated with FS^DSS^ with respect to controls after both acute intracolonic injection (1 h; Figure S2A) and repeated treatment (Day 10; Figure S2B).

*p<0.05 and **p<0.01 vs saline; ^*p*< 0.05 and ^^*p*<0.01 vs FS^CTR^.

### 3.2. Fecal supernatants from DSS-treated mice negatively affect epithelial colon organoids size but not their viability

**Figure 2.**
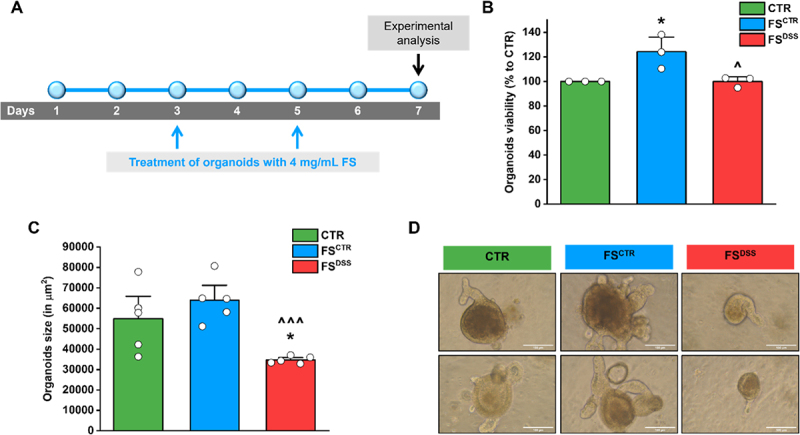
Effects of FS^CTR^ and FS^DSS^ on viability and growth ability of colon organoids. A) Colon organoids were treated on days 3 and 5 from the seeding (day 1) with 4 mg/mL FS^CTR^ and FS^DSS^, and experimental analysis were performed on day 7. B) MTT assay was performed on colon organoids following FS treatments to evaluate the effects of FS on their viability. C) Morphometric analysis was performed on colon organoids following FS treatments to assess the impact of FS on their growth ability. D) Representative images of organoids after treatment with FS (magnification: 20×; scale bar: 100 µm). Values represent the mean ± SEM of *n*=3 experiments for MTT assay and *n*=5 experiments for morphometric analysis. The analysis of variance was performed by one-way ANOVA followed by Bonferroni post hoc comparison.

At this point, we aimed to evaluate the effects of normal and altered fecal products on colon epithelium by using epithelial colon organoids. After a phenotypic characterization to ensure the presence of the main epithelial cell types inhabiting the colon, like stem cells, colonocytes, goblet cells and enteroendocrine cells (Figure S3), organoids were plated on day 1, exposed to 4 mg/mL FS^CTR^ and FS^DSS^ on days 3 and 5, and used for experimental analysis on day 7 as reported in Figure 2(A). Viability of organoids treated with FS^CTR^ was slightly increased, while no effects were observed under the treatment with FS^DSS^ (Figure 2(B)). Regarding morphological parameters, the treatment with FS^DSS^ consistently reduced the size of colon organoids compared to CTR and FS^CTR^ conditions (Figure 2(C)). Moreover, it is possible to appreciate a qualitative reduction of complexity in organoids exposed to FS^DSS^, as indicated in Figure 2(D). Overall, these data demonstrated that altered luminal products were able to impair the organoid growth ability without impairing their viability.

*p<0.05 vs CTR; ^*p*<0.05 and ^^^*p*<0.001 vs FS^CTR^.

### 3.3. Fecal supernatants from DSS-treated mice and conditioned medium from dysfunctional organoids drive hyperexcitability in DRG neurons

**Figure 3.**
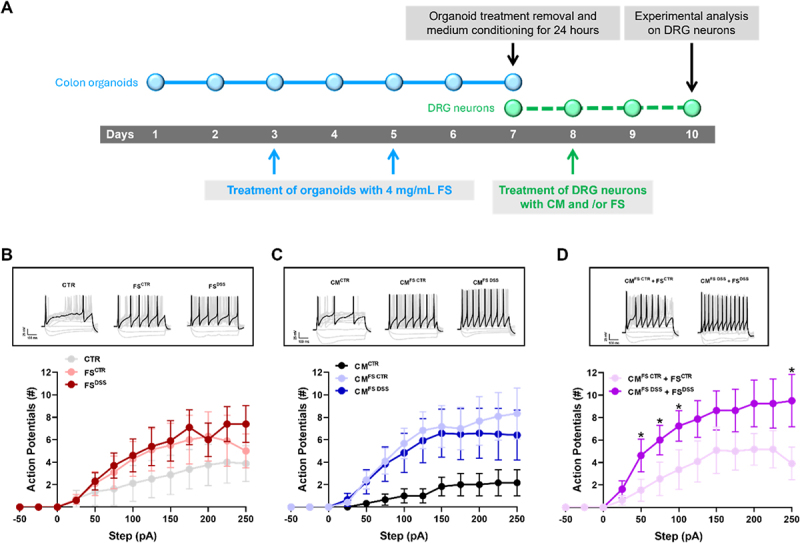
Electrophysiological analysis on DRG neurons exposed to FS and/or CM from colon organoids. A) DRG neurons were exposed for 48 hours to CM^CTR^, FS^CTR^, FS^DSS^, CM^FS CTR^, CM^FS DSS^, CM^FS CTR^ + FS^CTR^ and CM^FS DSS^ + FS^DSS^. Intrinsic excitability was measured in B) FS^CTR^ vs FS^DSS^, C) CM^FS CTR^ vs CM^FS DSS^ and D) CM^FS CTR^ + FS^CTR^ vs CM^FS DSS^ + FS^DSS^. Example traces of action potentials recordings were obtained in response to a +125 pA current. Values represent the mean ± SEM of 6–12 cells analyzed in *n*=4 experiments. The analysis of variance was performed by one-way ANOVA followed by Bonferroni post hoc comparison.

To investigate *in vitro* the effects of intestinal fecal and epithelial products on the excitability of DRG neurons, these latter were exposed for 48 hours to FS^CTR^ and FS^DSS^, conditioned media from FS^CTR^-treated organoids (CM^FS CTR^) and FS^DSS^-treated organoids (CM^FS DSS^), and the combination of CM^FS CTR^ + FS^CTR^ and CM^FS DSS^ + FS^DSS^, as shown in Figure 3(A). We first explored the passive properties of DRG neurons after the treatments and found no significant differences in membrane resistance (Figure S4A), resting membrane potential (Figure S4B) or membrane capacitance (Figure S4C). Interestingly, capacitance values indicated that neurons analyzed for each condition corresponded to those with small-medium diameter (from 21.32 ± 3.13 to 30.57 ± 3.87 pF), therefore to nociceptors, as previously reported.^43,44^ Regarding intrinsic excitability, no differences were found in the intrinsic excitability between the neurons treated with FS^CTR^ or FS^DSS^ (Figures 3(B) and S5), nor between those treated with CM^FS CTR^ or CM^FS DSS^ (Figures 3(C) and S5). In contrast, the analysis revealed that DRG neurons treated with CM^FS DSS^ + FS^DSS^ showed a significant increase in the number of evoked action potentials compared to the CM^FS CTR^ + FS^CTR^ group (Figures 3(D) and S5). Notably, no difference in firing threshold or viability was observed across experimental groups. (Figure S6 and S7). In summary, these findings reveal an integrated role of epithelial and microbial factors in modulating the excitability of DRG neurons *in vitro*.

*p<0.05 vs CM^FS CTR^ + FS^CTR^.

### 3.4. Fecal supernatants from DSS-treated mice and conditioned medium from dysfunctional organoids enhance c-Fos and CGRP expression in DRG neurons

**Figure 4.**
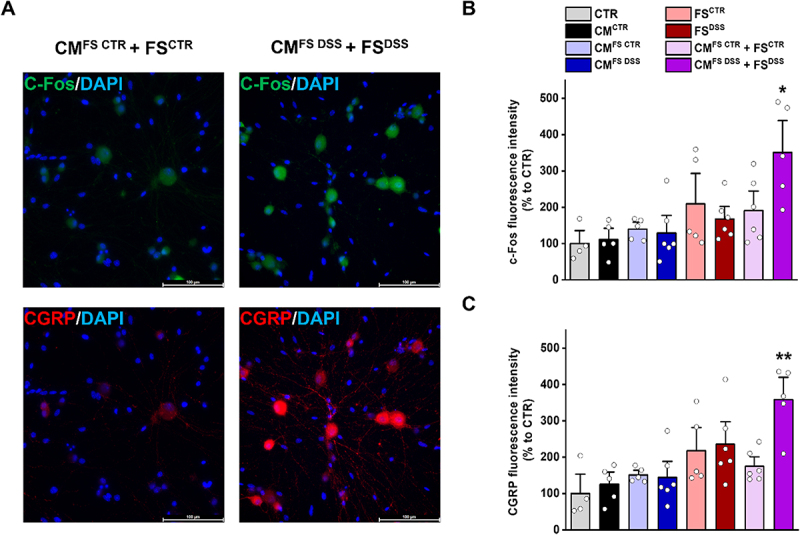
Immunofluorescence analysis for c-Fos and CGRP in DRG neurons exposed to FS and/or CM from colon organoids A) Representative images of DRG neurons stained for c-Fos (green) and CGRP (red) after 48 hours exposure to CM^FS CTR^ + FS^CTR^ and CM^FS DSS^ + FS^DSS^ (magnification: 40×; scale bar: 100 µm). Fluorescence intensity was measured for B) c-Fos and C) CGRP in all experimental groups. Values represent the mean ± SEM of *n*=4–6 different slides for each condition. The analysis of variance was performed by one-way ANOVA followed by Bonferroni post hoc comparison.

To further support the electrophysiological analysis, we evaluated the expression levels of c-Fos and CGRP in DRG neurons exposed for 48 hours to FS and/or CM by immunofluorescence. No significant differences were observed between FS^CTR^ and FS^DSS^ or CM^FS CTR^ and CM^FS DSS^, whereas the treatment with CM^FS DSS^ + FS^DSS^ induced a significant increase in the immunoreactivity for both c-Fos and CGRP compared to the CM^FS CTR^ + FS^CTR^ group (Figure 4). Representative images for all experimental conditions are reported in Figure S8. Increased c-Fos expression in the CM^FS DSS^ + FS^DSS^ group confirmed that dysbiosis condition and the correlated intestinal epithelial dysfunction led to an enhancement in neuronal excitability, resulting in peripheral neurogenic inflammation and increased pain transmission to the central ascending pathways as suggested by the increase in CGRP immunoreactivity.

*p<0.05 and **p<0.01 vs CM^FS CTR^ + FS^CTR^.

### 3.5. Fecal supernatants from DSS-treated mice and conditioned medium from dysfunctional organoids enhance c-Fos expression in different subpopulations of DRG neurons

**Figure 5.**
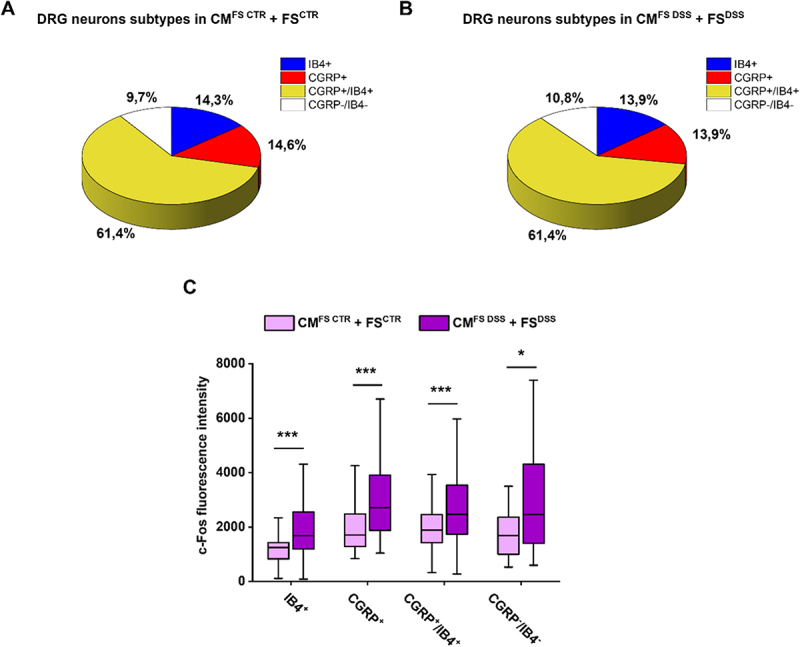
Assessment of c-Fos expression in different subpopulations of DRG neurons exposed to CM^FS CTR^ + FS^CTR^ and CM^FS DSS^ + FS^DSS^. Four subpopulations of DRG neurons with different proportions were identified in A) CM^FS CTR^ + FS^CTR^ and B) CM^FS DSS^ + FS^DSS^ groups, based on IB4-binding affinity and CGRP expression. C) The immunoreactivity for c-Fos was measured in IB4^+^, CGRP^+^, CGRP^+^/IB4^+^, and CGRP^−^/IB4^−^ subpopulations comparing the two experimental conditions. Lines represent the median within the box, the 25^th^ and 75^th^ percentiles at the ends of the box (interquartile range), and the error bars define the 25^th^ + 1.5 interquartile range and the 75^th^ + 1.5 interquartile range. The analysis of variance was performed by Kruskal-Wallis test followed by Dunn post hoc comparison.

To investigate which neuronal subpopulations were most involved in the neuronal response to conditions of altered luminal composition and associated epithelial dysfunction, further immunofluorescence analysis were carried out on DRG neurons treated for 48 hours with CM^FS CTR^ + FS^CTR^ and CM^FS DSS^ + FS^DSS^. We exploited CGRP expression and Isolectin B4 (IB4)-binding affinity to define four subpopulations of sensitive neurons: IB4^+^ (non-peptidergic C fibers), CGRP^+^ (peptidergic C fibers), CGRP^+^/IB4^+^ (Aδ fibers) and CGRP^−^/IB4^−^ (Aβ fibers). In both experimental conditions, the proportions of subpopulations were very similar, and the most represented was the CGRP^+^/IB4^+^ (~60%); IB4^+^ and CGRP^+^ each accounted for around 14% of the total, whereas CGRP^−^/IB4^−^ were around 10% of the total (Figures 5(A,B)). Interestingly, c-Fos signal was significantly higher in all the subpopulations of DRG neurons treated with CM^FS DSS^ + FS^DSS^ (Figure 5(C)), suggesting that all sensory fibers examined are involved in dysbiosis-related visceral hypersensitivity.

*p<0.05 and ***p<0.001.

### 3.6. RNA sequencing reveals a metabolic injury and an altered autocrine/paracrine signaling in organoids exposed to fecal supernatants from DSS-treated mice

**Figure 6.**
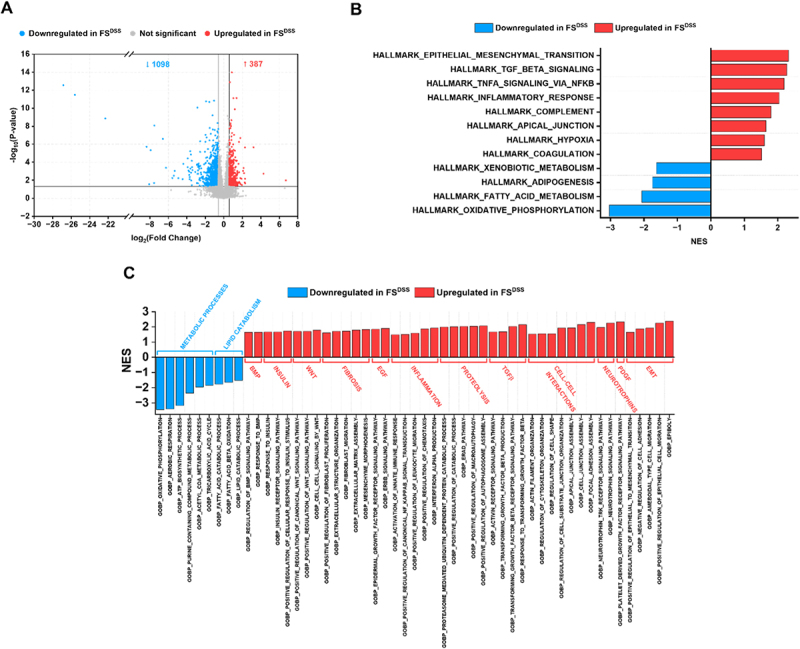
Analysis of colon organoid transcriptomic profile under FS^CTR^ and FS^DSS^ treatments. A) Volcano plot shows the genes that are differentially expressed in organoids in the comparison FS^DSS^ vs FS^CTR^. Gene set enrichment analysis (GSEA) was performed in B) Hallmark and C) GO:BP datasets to identify which pathways emerge in the comparison FS^DSS^ vs FS^CTR^. RNA sequencing analysis was conducted on *n*=4 samples for each condition. Genes with *p*<0.05 and Log_2_(FC)≥0.58 or ≤-0.58 were considered as significant DEGs. Pathways significantly modulated were selected according to the adjusted *p*<0.05.

We performed an RNA sequencing analysis on colon organoids treated with fecal supernatants to find epithelial alterations that might be partially responsible for neuronal hyperexcitability. Focusing on the comparison among FS^DSS^ vs FS^CTR^, we identified 1485 significant differentially expressed genes (DEGs): 387 upregulated genes (*p*<0.05 and Log_2_(Fold Change (FC)) ≥0.58) and 1098 downregulated genes (*p*<0.05 and Log_2_(FC) ≤–0.58) (Figure 6(A)). All the detected genes are listed in Supplementary Dataset 1 at https://doi.org/10.17632/4k5yfp4nxd.1.

To identify the enriched gene sets that are associated with FS^DSS^ group, a gene set enrichment analysis (GSEA) was conducted based on Hallmark datasets and modulated pathways of interest are reported in Figure 6(B). Gene sets of HALLMARK_OXIDATIVE_PHOSPHORYLATION, HALLMARK_FATTY_ACID_METABOLISM, HALLMARK_ADIPOGENESIS and HALLMARK_XENOBIOTIC_METABOLISM negatively correlated with FS^DSS^ group. Conversely, gene sets related to HALLMARK_COAGULATION, HALLMARK_HYPOXIA, HALLMARK_APICAL_JUNCTION, HALLMARK_COMPLEMENT, HALLMARK_INFLAMMATORY_RESPONSE, HALLMARK_TNFA_SIGNALING_VIA_NFKB, HALLMARK_TGF_BETA_SIGNALING, and HALLMARK_EPITHELIAL_MESENCHYMAL_TRANSITION were enriched in organoids treated with FS^DSS^. To validate and further explore these findings, we conducted another GSEA in Gene Ontology: Biological Processes (GO:BP) datasets and several relevant pathways emerged as differentially modulated under FS treatments (Figure 6(C)). Consistent with the GSEA analysis in Hallmark, pathways related to metabolic processes and lipid catabolism negatively correlated with FS^DSS^ group, while gene sets related to inflammation, fibrosis, proteolysis, transforming growth factor beta (TGF-β), cell-cell interactions and epithelial-mesenchymal transition (EMT) processes were enriched in FS^DSS^ group. Importantly, several pathways related to bone morphogenetic protein (BMP), insulin, Wnt, epidermal growth factor (EGF), TGF-β, neurotrophins and platelet-derived growth factor (PDGF) modules were significantly enriched in the FS^DSS^ group, suggesting an altered autocrine/paracrine signaling in FS^DSS^-treated organoids (all the modulated gene sets resulting from GSEA in Hallmark and GO:BP are listed in Supplementary Dataset 2 and 3 at https://doi.org/10.17632/4k5yfp4nxd.1).

Thus, we focused our attention on some gene families that were differentially expressed in organoids under the treatment with FS^DSS^ and FS^CTR^. A large cluster of genes coding for the proteins involved in the mitochondrial electron transport chain was downregulated in organoids treated with FS^DSS^ (Figure S9), confirming the negative correlation between FS^DSS^ group and gene sets related to HALLMARK_OXIDATIVE_PHOSPHORYLATION, GOBP_OXIDATIVE_PHOSPHORYLATION, GOBP_AEROBIC_RESPIRATION, GOBP_ATP_BIOSYNTHETIC_PROCESS, GOBP_PURINE_CONTAINING_COMPOUND_METABOLIC_PROCESS, GOBP_ACETYL_COA_METABOLIC_PROCESS, and GOBP_TRICARBOXYLIC_ACID_CYCLE among others (Figures 6(B,C)). Notably, the mitochondrial genome encodes 13 genes regulating ATP production and all 13 were significantly reduced in FS^DSS^ group (Figure S9).

**Figure 7.**
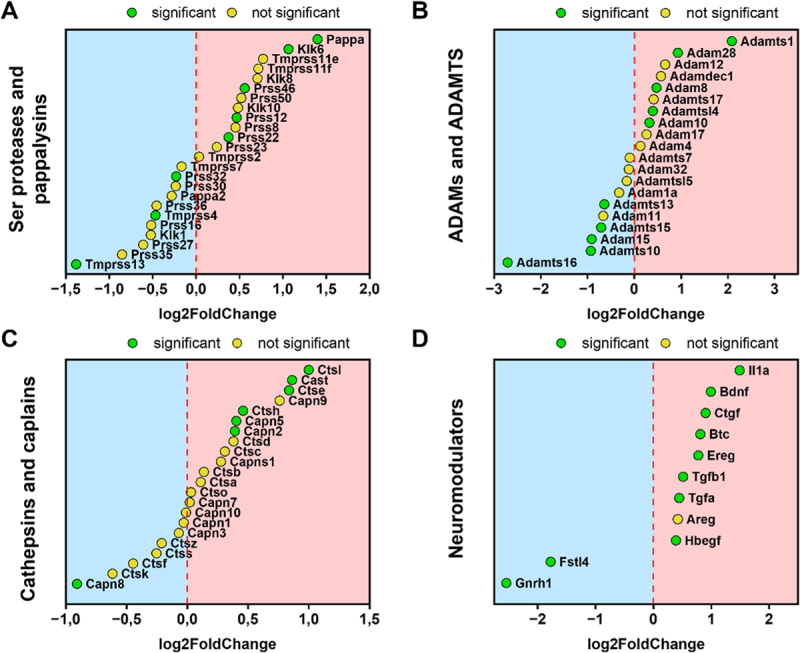
Expression of relevant genes in colon organoids under FS^CTR^ and FS^DSS^ treatments. The analysis was conducted for genes coding for A) serine proteases and pappalysins, B) ADAMs and ADAMTS, C) cathepsins and calpains, and D) neuromodulators. Genes in the red area were upregulated in FS^DSS^ while genes in the blue area were downregulated in FS^DSS^.

Furthermore, a severe disturbance of the proteolytic balance was confirmed in FS^DSS^ group, given the positive correlation with GOBP_PROTEASOME_MEDIATED_UBIQUITIN_DEPENDENT_PROTEIN_CATABOLIC_PROCESS, GOBP_POSITIVE_REGULATION_OF_CATABOLIC_PROCESS, GOBP_ERAD_PATHWAY, GOBP_POSITIVE_REGULATION_OF_MACROAUTOPHAGY, and GOBP_POSITIVE_REGULATION_OF_AUTOPHAGOSOME_ASSEMBLY among others (Figure 6(C)). Genes coding for several types of proteases were effectively deregulated in organoids exposed to FS^DSS^. The expression of genes coding for serine proteases (*Klk6*, *Prss46*, *Prss12*, and *Prss22*) and pappalysin (*Pappa*) was increased in organoids exposed to FS^DSS^, while *Prss32*, *Tmprss4* and *Tmprss13* were downregulated (Figure 7(A)). Regarding A Disintegrin And Metalloprotease (ADAM) and A Disintegrin And Metalloprotease with thrombospondin motifs (ADAMTS) families, our analysis revealed that *Adamts1*, *Adam28*, *Adam8*, *Adamtsl4*, and *Adam10* were upregulated in FS^DSS^ group whereas *Adamts13*, *Adamts15*, *Adam15*, *Adamts10*, and *Adamts16* were downregulated in the same group (Figure 7(B)). While there seemed to be a balance regarding serine proteases, pappalysins, ADAM, and ADAMTS between FS^CTR^ and FS^DSS^, the expression of genes coding for cathepsins and calpains was unbalanced toward the FS^DSS^ group. Indeed, *Ctsl*, *Cast*, *Ctse*, *Ctsh*, *Capn5*, and *Capn2* were upregulated in organoids treated with FS^DSS^, while only *Capn8* resulted downregulated (Figure 7(C)). Furthermore, although several genes coding for serpins (serine protease inhibitors) seemed to be upregulated in FS^DSS^ (*Serpinb2, Serpinb8, Serpine2, Serpine1, Serpinb5*) (Figure S10), no clear results were obtained about other protease inhibitors.

Statistical significance was set to *p*<0.05.

Lastly, we identified a group of deregulated genes encoding proteins that may also be involved in the modulation of neuronal sensitivity, that includes diverse EGF receptor (EGFR) ligands, cytokines and autocrine modulators (Figure 7(D)). The gene (*Il1a*) coding for the interleukin-1 alpha (IL-1α) was the most upregulated in FS^DSS^-treated organoids. Another important pain mediator is the brain-derived neurotrophic factor (BDNF), whose gene *Bdnf* was 2-fold upregulated in FS^DSS^-treated organoids. Regarding EGFR ligands, *Ctgf*, *Btc*, *Ereg*, *Tgfa*, and *Hbegf*, respectively coding for connective tissue growth factor (CTGF), betacellulin, epiregulin (EREG), transforming growth factor alpha (TGF-α), and heparin-binding EGF-like growth factor (HB-EGF), were significantly upregulated in organoids exposed to FS^DSS^. A further upregulated gene in FS^DSS^ group was *Tgfb1*, coding for TGF-β1. By contrast, we identified two downregulated genes in FS^DSS^ organoids which are *Fstl4* and *Gnrh1*, coding for follistatin-like protein 4 (FSTL4) and gonadotropin releasing hormone (GnRH). Overall, all these deregulated genes reinforce the idea of an altered autocrine/paracrine signaling as demonstrated by the GSEA.

### 3.7. Fecal supernatants and conditioned media from colon organoids display different metabolic signatures across the experimental groups

**Figure 8.**
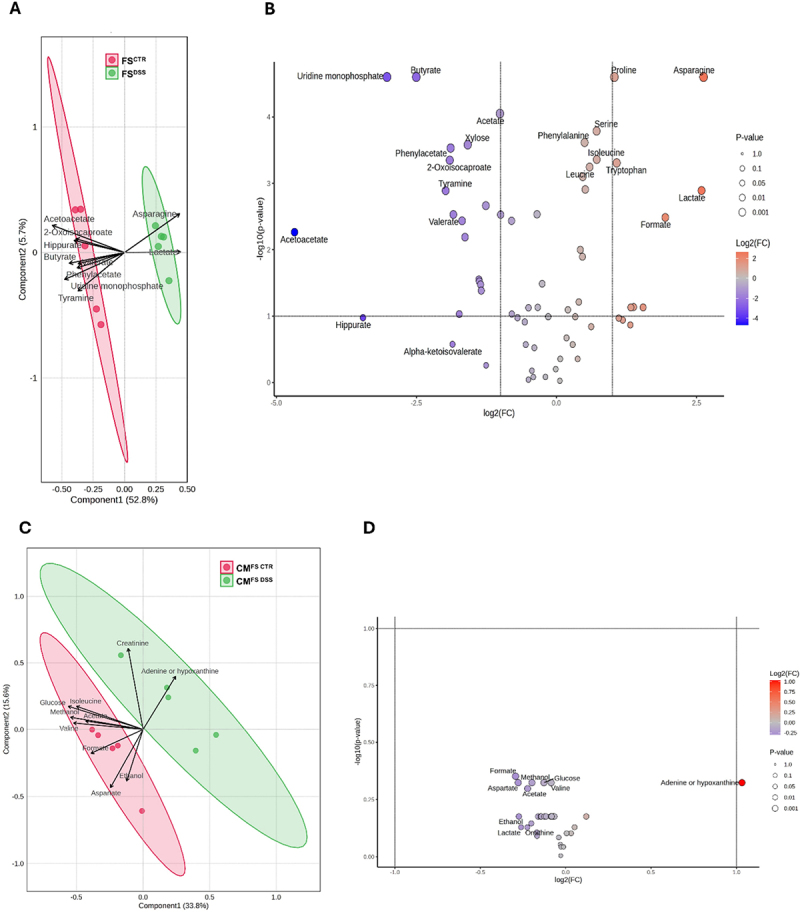
Metabolomic characterization of fecal supernatants and conditioned media from organoids. Metabolomic differences in FS^DSS^ vs FS^CTR^ and CM^FS DSS^ vs CM^FS CTR^ comparisons were reported respectively in A, C) PLS-DA and B, D) volcano plots. The analysis was conducted on *n*=5 samples for each condition. Adjusted *p*<0.1 was considered statistically significant.

To further investigate the mechanisms underlying visceral hypersensitivity, we carried out metabolomic assessments of FS^CTR^ and FS^DSS^, along with CM^FS CTR^ and CM^FS DSS^. In fecal supernatants, the metabolomic profile was distinctly different across the experimental groups as shown by the PLS-DA plot, which depicts clear separation of each group indicating a metabolomic shift in FS^DSS^ vs FS^CTR^ comparison (Figure 8(A)). This is further emphasized by the volcano plot which depicts the concentration of the significantly modulated metabolites (Figure 8(B) and Supplementary Dataset 4 at https://doi.org/10.17632/4k5yfp4nxd.1; FDR *q*<0.1). Among metabolites modulated, adenine/hypoxanthine and SCFAs acetate, butyrate, propionate and valerate were significantly decreased in FS^DSS^ when compared to FS^CTR^, whereas formate and lactate showed an opposite trend. In organoid conditioned media, there was a relevant shift in metabolic signature (Figure 8(C)). Nevertheless, no significant differences were observed between CM^FS DSS^ and CM^FS CTR^ as depicted in the volcano plot (Figure 8(D) and Supplementary Dataset 5 at https://doi.org/10.17632/4k5yfp4nxd.1; FDR *q* < 0.1), even though adenine/hypoxanthine was the most upregulated metabolite in CM^FS DSS^.

## 4. Discussion

The present work reports for the first time the contribution of the intestinal epithelium in modulating intestinal sensory circuitries in the presence of fecal products by using an innovative *in vitro* model consisting of murine epithelial colon organoids and primary DRG neurons.

Although chronic pain of gastrointestinal origin affects more than 20% of world’s population^45^, effective therapies are still not available due to its complexity and heterogeneity^2^. In many cases, visceral hypersensitivity persists even after the remission of the organic disease that caused it. Among the responsible for pain onset and persistence in the gut, there is dysbiosis of microbiota^5,15,46^. However, it is not clear whether the effect is due directly to microbiota or other host components, such as intestinal epithelium, that represents the first site of interaction between the microbiota and the host and may modulate the pain signaling through the gut-brain axis. We used a mouse model of visceral abdominal pain associated with post-inflammatory dysbiosis caused by DSS administration, that resembles the intestinal damage observed in patients with ulcerative colitis, including epithelial alterations^47–49^ and important changes in microbial diversity.^50–52^

Through *in vivo* studies, we first demonstrated that the intracolonic instillation of FS^DSS^ is enough to induce a long-lasting visceral hypersensitivity in naïve mice, indicating that the luminal products, irrespective to the presence of microbes, regulate intestinal sensitivity. This hypothesis is further supported by several studies demonstrating that the intracolonic infusion of fecal supernatants from IBS patients displayed pro-nociceptive effects in recipient rodents.^53–57^ NMR analysis showed that FS^DSS^ exhibited a negative correlation with the abundance of some SCFAs, such as butyrate, acetate, propionate, and valerate, and a positive correlation with lactate and formate. Even if there is no relevant data about valerate and formate in visceral pain, most of SCFAs may modulate visceral sensitivity. In fact, butyrate administration has been shown to decrease visceral pain and discomfort in healthy volunteers^58^ and patients with IBS or other gastrointestinal disorders.^59–61^ Moreover, butyrate-producing microorganisms are reduced in IBS patients.^62^ Levels of butyrate, acetate, propionate were also found to be decreased in IBD patients, while lactate abundance was increased,^63^ being in line with our data. Similarly, in rodent models, reduction in the abundance of butyrate-producing microbes was involved in the onset of visceral hypersensitivity^64^ and intestinal administration of butyrate alleviates visceral hyperalgesia in IBS-like conditions.^65,66^ Other evidence comes from studies on germ-free mice, which display visceral hypersensitivity,^67,68^ where butyrate, acetate and propionate are significantly reduced.^69^ Nevertheless, several studies have showed contradictory results, reporting that butyrate enemas induced or prolonged visceral hypersensitivity in rats.^70–73^ Another study demonstrated that capsaicin-evoked calcium responses were increased in naïve DRG neurons incubated with butyrate and propionate.^15^ Therefore, more studies are needed to better elucidate the role of SCFAs in visceral pain modulation. However, we observed no difference in the intrinsic excitability of DRG sensory neurons exposed to the different FS treatments, so we can exclude that SCFAs as well as other microbial products by itself can affect visceral sensitivity in our conditions. Instead, this evidence proved that other partners within the gut may transduce the pain message delivered by the microbiota.

In this regard, we employed murine epithelial colon organoids to investigate the role of intestinal epithelium in dysbiosis-associated visceral hypersensitivity. Although the treatment with both FS^CTR^ and FS^DSS^ did not impair organoid viability, we noticed that organoids exposed to FS^DSS^ displayed a marked reduction in size and structural complexity. Such findings are in agreement with d’Aldebert et al. who reported that colon organoids derived from IBD patients had a significantly smaller size and complexity than control organoids,^74^ resembling the condition we reproduced through the exposure of murine colon organoids to the FS from colitis animals. Therefore, we demonstrated that dysbiosis drives some typical epithelial dysfunctions associated with gastrointestinal pathologies. In this regard, RNA sequencing analysis showed that FS^DSS^ treatment caused a significant reduction in the expression of genes involved in oxidative phosphorylation, mitochondrial electron transport chain and lipid catabolism, highlighting potential mitochondrial dysfunctions and cellular respiration deficits that could be responsible for the reduced growth observed in organoids. Noteworthy, mitochondrial dysfunctions involving the intestinal epithelium have been reported in IBD patients^75–79^ as well as in a DSS colitis model.^80^ Since butyrate is the preferred energy source of colonic epithelial cells for generating ATP,^81,82^ its lower abundance in FS^DSS^ might explain the hampered metabolism and growth ability of FS^DSS^-treated organoids. Moreover, hypoxanthine levels were also low in FS^DSS^. Low levels of hypoxanthine were also reported in stool of IBS patients^83^ and colon of DSS-treated mice.^84^ Gut epithelial cells preferentially use the salvage pathway to sustain their metabolism and hypoxanthine provides a readily available substrate,^84–86^ supporting that hypoxanthine starvation in luminal environment represents another cause of metabolic dysfunctions in the gut epithelium, thus in FS^DSS^-treated organoids. On the other hand, the epithelium stressed by dysbiosis might not be able to adequately absorb these energy resources. Indeed, our RNA sequencing revealed a significant downregulation of genes encoding solute carriers involved in nutrient uptake in FS^DSS^-treated organoids, such as *Slc28a2* (Concentrative nucleoside transporter 2), *Slc16a3* (Monocarboxylate transporter 4), *Slc15a1* (Peptide transporter 1), *Slc43a1* (Large neutral amino acids transporter small subunit 3), and *Slc50a1* (Sugar transporter SWEET1) (Supplementary Dataset 1 at https://doi.org/10.17632/4k5yfp4nxd.1).

In addition to structural changes in their metabolism and sensitivity to external stimuli, FS^DSS^-treated colon organoids underwent changes in the gene expression of signaling factors that once released by the epithelium could influence the activity of neighboring terminals of DRG sensory neurons (responsible for the transmission of sensory information, including pain, from the periphery to the central nervous system).

To corroborate this hypothesis, we studied the impact of dysbiosis on epithelium-to-neuron signaling involved in visceral sensitivity regulation, by implementing the organoid system with murine primary DRG neurons. DRG neurons treated with CM^FS DSS^ + FS^DSS^ displayed higher intrinsic excitability compared to those exposed to CM^FS CTR^ + FS^CTR^, while no differences were observed between DRG neurons treated with only FS^CTR^ or FS^DSS^, nor between those treated with only CM^FS CTR^ or CM^FS DSS^. These results indicated that FS^DSS^ likely induces visceral hypersensitivity in mice collaborating with the intestinal epithelium, revealing for the first time an integrated involvement of microbiota and epithelium in the regulation of visceral sensitivity. Our results partially contradict some previous evidence which showed that only supernatants from the stool, but not from colon tissue, of vancomycin-treated mice excited DRG neurons through the protease-activated receptor 2 (PAR-2).^13^ However, in a previous work, epithelial products derived from IBD patients have been reported to induce DRG hyperexcitability via a tumor necrosis factor alpha (TNF-α)-mediated mechanism.^87^ Similarly, exposure of DRG neurons to colonic supernatants from DSS mice increased intracellular calcium through the transient receptor potential vanilloid 1 receptor (TRPV1).^15^ All these paradigms, although different in approach, confirm an implication of both microbiota and epithelium dysfunction in visceral hypersensitivity persistence through different mechanisms.

Furthermore, CM^FS DSS^ + FS^DSS^ was found to increase both c-Fos, a molecular marker of neuronal activity,^88,89^ and CGRP, a mediator involved in pain signaling^90^ and neurogenic inflammation.^4,28,29^ Noteworthy, CGRP release from nociceptors in the gut represents a defense mechanism against pathogens.^91^ Besides, it was demonstrated that CGRP production in response to microbial metabolites is higher in cultured DRG neurons from germ-free mice, confirming the existence of a vicious circle between dysbiosis and pain.^68^ Furthermore, by analyzing DRG-derived peptidergic C (CGRP^+^),^28^ non-peptidergic C (IB4^+^),^39^ Aδ (IB4^+^/CGRP^+^) and Aβ fibers (IB4^-^/CGRP^-^)^40^ separately, we found a significantly higher c-Fos signal in all four subpopulations treated with CM^FS DSS^ + FS^DSS^ compared to those treated with CM^FS CTR^ + FS^CTR^.

In addition to the products contained in FS^DSS^, DRG neuron hyperexcitability might be ascribed to the altered expression of some genes encoding signaling proteins in FS^DSS^-treated colon organoids, as detected by RNA sequencing. First, we reported a significant alteration in the proteolytic balance of organoids treated with FS^DSS^. Elevated proteolytic activity mediates visceral hypersensitivity, especially in IBS and IBD patients.^13,92–95^ This effect could be ascribed to the crucial role played by proteases in the epithelial-neuronal communication through activation of PARs, particularly PAR2, highly expressed in epithelial cells and sensory neurons.^92,95,96^ Our data showed an imbalance in the expression of some calpains and cathepsins, with *Ctsl*, *Ctse*, *Ctsh*, *Capn5*, and *Capn2* upregulated in FS^DSS^ and only *Capn8* downregulated. Cathepsins^97,98^ as well as calpain 2^99^ were shown to be involved in pain mechanisms, highlighting the potential of this class of proteases as a target for visceral pain treatment. Second, FS^DSS^ group displayed dysregulated expression of some neuromodulators that could play an important role in the context of epithelial-neuronal pain signaling, including upregulation of genes encoding IL-1α, TGF-β1, EGFR ligands, BDNF, and downregulation of genes encoding FSTL4 and GnRH. IL-1α expression is increased in IBD patients^100–102^ and its neutralization through a specific antibody significantly ameliorated the ileitis course in a mouse model of IBD by correcting microbial dysbiosis.^101^ Given that IL-1 receptor was found to be highly expressed in both mouse and human nociceptors,^103^ colonic IL-1α might modulate visceral sensitivity. Concerning TGF-β1, evidence has shown enhanced levels of this protein in blood and mucosa of IBD patients.^104–106^ Interestingly, beyond its role in tissue fibrosis and immune regulation,^107^ TGF-β1 is positively involved in inflammatory pain signaling.^108–111^ Mechanisms of action could involve sensitization of TRPV1 or augmented production of substance P in sensory neurons,^109,112,113^ demonstrating a potential role for TGF-β1 in neuronal hyperexcitability as well. Upregulation of genes coding for EGFR ligand (*Ctgf*, *Btc*, *Ereg*, *Tgfa*, and *Hbegf*) in FS^DSS^-treated organoids provided further evidence about the role of gut epithelial dysfunction related to dysbiosis in driving visceral hyperalgesia. Several studies have demonstrated that EGFR may be broadly expressed in DRG neurons and spinal cord as well as immune and supportive cells relevant to pain^114–118^, highlighting its contribution to pain signaling. Although EREG is the most investigated EGFR ligand in the context of pain,^115,116,119^ EGF and HB-EGF administrations were shown to evoke pain responses as well.^116,120^ Accordingly, evidence suggests that the targeting of EGFR may alleviate pain in humans^121–124^ and animals.^115–117,125,126^ However, no reports exist about EGFR targeting in visceral pain conditions, making EGF signaling an attractive therapeutic target for painful gastrointestinal diseases. *Bdnf* was another upregulated mRNA in colon organoids exposed to FS^DSS^. Concurrently, it is worth noting that FS^DSS^-treated organoids displayed a strong downregulation of *Fstl4* mRNA, coding for a protein that negatively regulates BDNF maturation, thus its biological effect.^127^ It is well documented that BDNF and other neurotrophins are involved in chronic pain establishment,^128,129^ especially in abdominal pain related to IBS.^130–135^ However, only one study reported that blocking the BDNF signaling ameliorated visceral hypersensitivity in an IBS-like rat model.^136^ Moreover, no therapeutic strategies to modulate epithelial-derived BDNF have been developed so far. Also, a downregulation of *Gnrh1* was observed in organoids exposed to FS^DSS^. Although GnRH is mainly produced and released by hypothalamic neurons, this hormone can be produced by both small and large intestine.^137^ GnRH analogues were reported to be effective in relieving pain associated with endometriosis,^138–144^ reducing abdominal pain in premenopausal- or menstrual cycle-related IBS^140,141^ and alleviating IBS symptoms in rats.^145^ Finally, IgM antibodies against GnRH and its receptors were found to be elevated in serum of IBS patients, suggesting that its signaling is strongly involved in the IBS symptomatology.^146,147^ Overall, transcriptomic analysis demonstrated that colon organoids were able to sense different signals depending on the FS treatment, that they translate into different transcriptomic signatures. In particular, pro-excitatory signals emerged from colon epithelial organoids treated with FS^DSS^, which deserve to be further investigated from a pharmacological point of view. In this regard, it is important to consider that changes in the secretome of organoids treated with FS^DSS^ are not enough to elicit a response in neurons by themselves, as the combination with FS^DSS^ stimulus is needed to observe an increase in the excitability of DRG neurons. Yet, the increase in the excitability of DRG neurons under dysbiosis conditions might be due to the loss of an inhibitory signaling exerted by epithelium on sensory neurons, rather than to a direct excitatory stimulus. It is important to emphasize that our work extends beyond existing literature,^13,15,87,148^ by revealing long-term neuroplastic adaptations. Future studies will aim at examining how chronic exposure shapes subsequent responsiveness to acute secreted factors, and which is the “timing threshold” to modify the phenotype of sensory neuron excitability. This aspect could also have a high relevance in the setting of the therapeutic regimen.

In conclusion, we demonstrated that microbe-free fecal supernatants from mice with post-inflammatory dysbiosis induced visceral hypersensitivity when intracolonically instilled into recipient mice. This phenomenon is strongly mediated by the intestinal epithelium, as emerged from the analysis conducted through an innovative *in vitro* approach based on colon epithelial organoids and DRG neurons. This model, besides representing a reliable and clinically translatable platform for the screening of new therapeutic intervention, has been useful to reveal some intriguing epithelium-to-neuron signals to be considered as potential pharmacological targets for developing novel therapeutics for visceral pain. Building on the insights from this study, future research should focus on dissecting the molecular mechanisms underlying epithelial-neuronal communication in the context of dysbiosis-induced visceral hypersensitivity. Key priorities consist in identifying the specific roles of misregulated epithelial-derived factors through targeted strategies and isolating specific microbial- or epithelial-derived products involved in the signaling to sensory neurons. In parallel, *in vivo* validation using pharmacological and biotechnological strategies (including gnotobiotic models) will be critical in establishing causal links between epithelial signals and visceral pain. The inclusion of patient-derived colon organoids and induced pluripotent stem cell-derived neurons will enhance the translational relevance.

## Supplementary Material

Margiotta_et_al_Supplementary_Material _R2.docx

## Data Availability

The RNA-seq data, including both raw FASTQ files and the resulting raw count matrices generated using Salmon, have been deposited in the NCBI Gene Expression Omnibus (GEO) database under accession number GSE294757. The other data that support the findings of this study are openly available in Mendeley Data at https://doi.org/10.17632/4k5yfp4nxd.1.
