# Supplementary material for: Gut microbiota dysbiosis affects intestinal sensitivity through epithelium-to-neuron signaling: novel insights from a colon organoid-based model to improve visceral pain therapy
Source: Gut Microbes. 2025 Sep 3;17(1):2547029. doi: 10.1080/19490976.2025.2547029 (PMC12413070; doi:10.1080/19490976.2025.2547029)
Supplement: Margiotta_et_al_Supplementary_Material _R2.docx [file KGMI_A_2547029_SM9060.docx]

**Gut microbiota dysbiosis affects intestinal sensitivity through epithelium-to-neuron signaling: novel insights from a colon organoid-based model to improve visceral pain therapy**

Francesco Margiotta^1, #^, Elena Lucarini^1, #^, Alessandra Toti^1,^ *, Lorenzo Curti^1^, Alessio Masi^1^, Tommaso Mello^2^, Gwenaelle Le Gall^3^, Gianluca Mattei^4^, Alberto Magi^5^, David Vauzour^3^, Guido Mannaioni^1^, Lorenzo Di Cesare Mannelli^1^, Carla Ghelardini^1^

*^1^Department of Neuroscience, Psychology, Drug Research and Child Health - NEUROFARBA - Pharmacology and Toxicology Section, University of Florence, Florence, Italy.*

*^2^Department of Clinical and Experimental Biomedical Sciences "Mario Serio", University of Florence, Florence, Italy.*

*^3^Norwich Medical School, University of East Anglia, Norwich NR4 7TJ, United Kingdom.*

*^4^Department of Health Sciences Clinical Pharmacology and Oncology Unit, Viale Pieraccini 6, 50139, Florence University of Florence.*

*^5^Department of Information Engineering, University of Florence, Florence, Italy.*

** Correspondence:* [*alessandra.toti@unifi.it*](mailto:alessandra.toti@unifi.it)

*# These authors contributed equally to this work*

# SUPPLEMENTARY MATERIAL


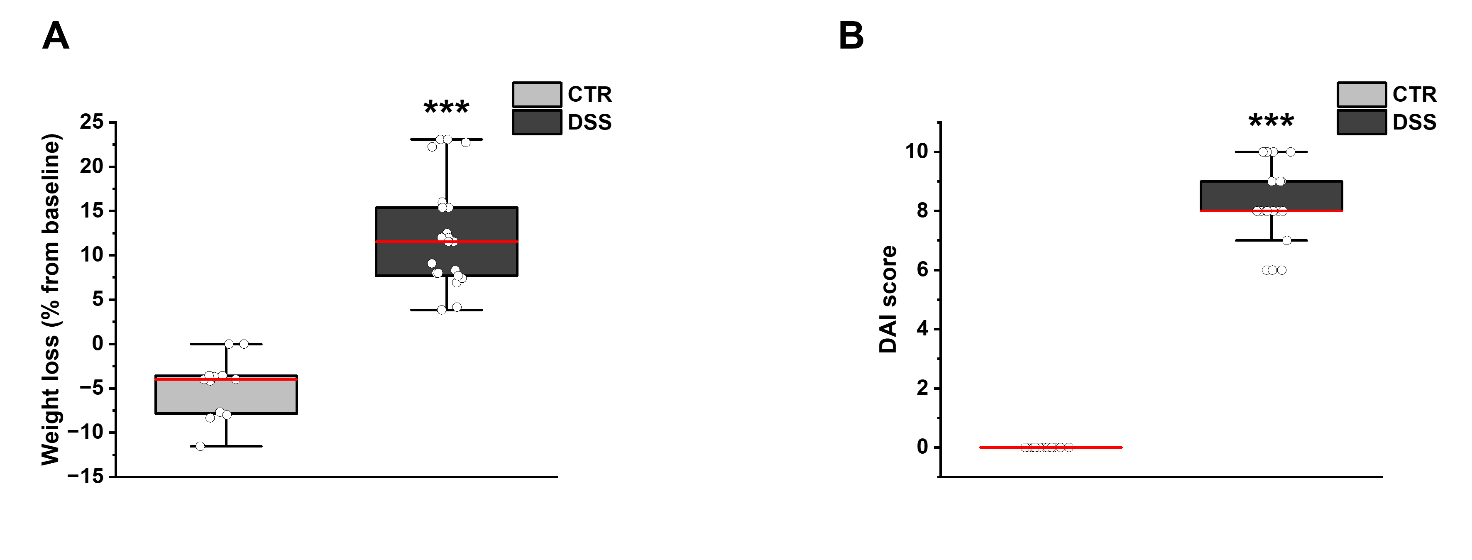


**Figure S1. Disease severity in DSS-treated mice.** Mice were treated with 2.5% (w:v) DSS for 5 days, followed by DSS-free tap water in the next 3 days. A) Weight loss and B) Disease Activity Index (DAI) score were assessed at the end of the treatment (day 8). Lines represent the median within the box (red), the 25th and 75th percentiles at the ends of the box (interquartile range; black), and the error bars define the 25th + 1.5 interquartile range and the 75th + 1.5 interquartile range. The analysis of variance was performed by Kruskal-Wallis test followed by Dunn post hoc comparison. ***P<0.001 vs CTR.


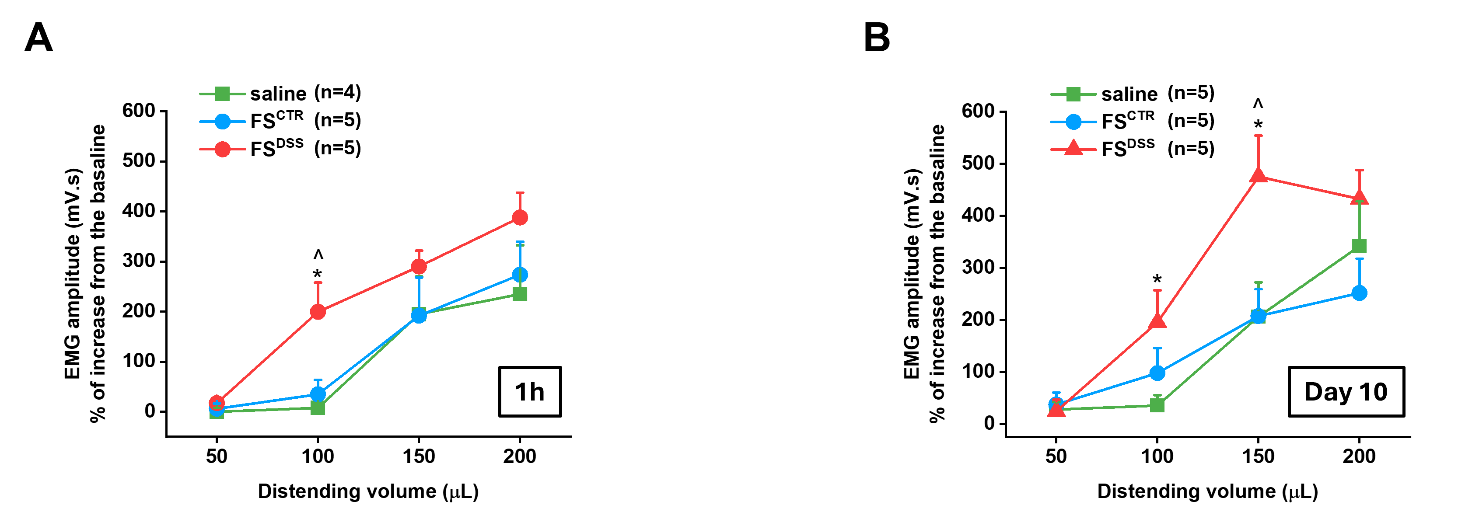


**Figure S2. Effects of the intracolonic injection of FS^CTR^ and FS^DSS^ on visceral sensitivity of naïve mice - assessment of visceromotor response to colorectal distension.** FS^CTR^ and FS^DSS^ (300 µL 100 mg/mL) were injected in naïve animals once daily for 4 consecutive days and visceral sensitivity was assessed by measuring the EMG amplitude (mV.s) of the abdominal contraction (visceromotor response; VMR) induced by colorectal distension (50-200 µL) A) 1 hour after the first FS injection and B) 7 days after the last FS injection. Values represent the mean ± SEM of each experimental group. The analysis of variance was performed by One-way ANOVA followed by Bonferroni post hoc comparison. *P<0.05 vs saline; ^P<0.05 vs FS^CTR^.


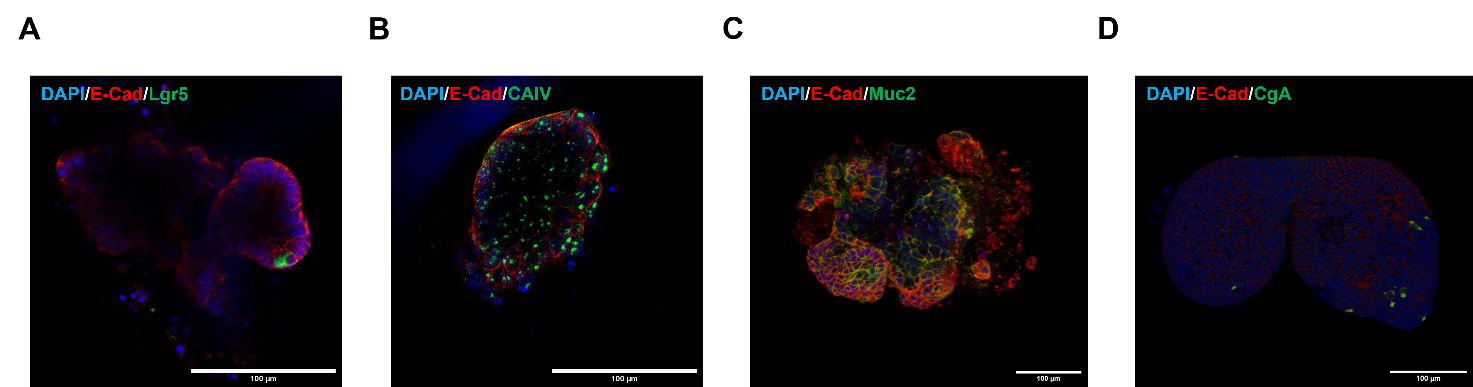


**Figure S3. Phenotypic characterization of mature colon organoids.** Colon organoids cell type diversity was assessed by immunofluorescence analysis through confocal microscopy. In all acquisitions E-cadherin (E-Cad, red) labels cell-to-cell junctions and 4′,6-diamidino-2-phenylindole (DAPI, blue) labels nuclei. A) Leucine-rich repeat-containing G-protein coupled receptor 5 (Lgr5, green) staining for intestinal stem cells; B) Carbonic anhydrase IV (CAIV, green) staining for colonocytes; C) Mucin 2 (Muc2, green) staining for goblet cells; D) Chromogranin A (CgA, green) staining for enteroendocrine cells. Magnification: 20X; scale bar: 100 µm.


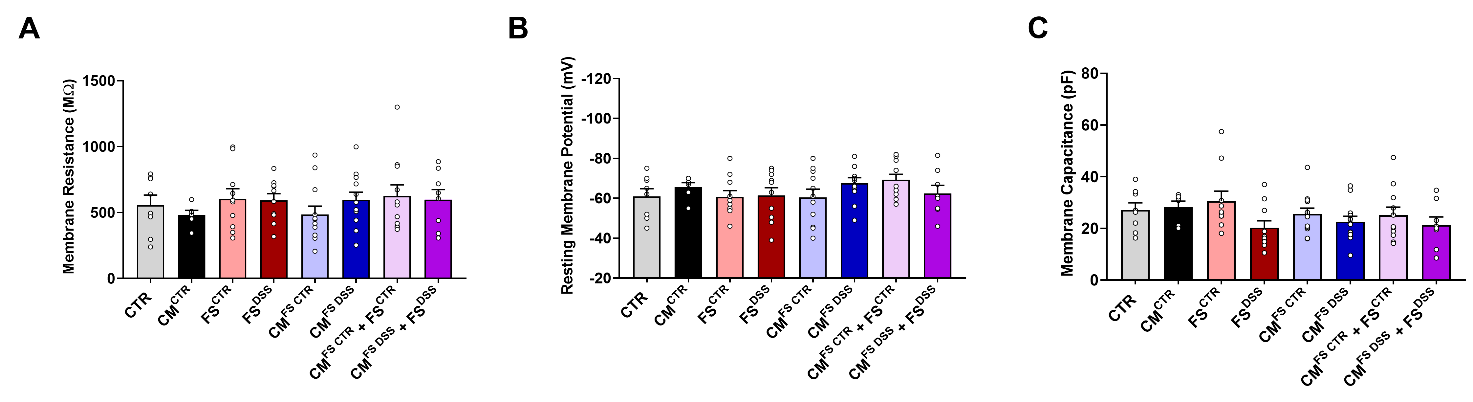


**Figure S4. Analysis of passive properties of DRG neurons exposed to FS and/or CM from colon organoids.**

DRG neurons were exposed for 48 hours to CM^CTR^, FS^CTR^, FS^DSS^, CM^FS CTR^, CM^FS DSS^, CM^FS CTR^ + FS^CTR^ and CM^FS DSS^ + FS^DSS^ and passive properties, such as A) membrane resistance, B) resting membrane potential and C) membrane capacitance, were measured. Values represent the mean ± SEM of 6-12 cells analyzed in n=4 experiments. The analysis of variance was performed by One-way ANOVA followed by Bonferroni post hoc comparison.

**
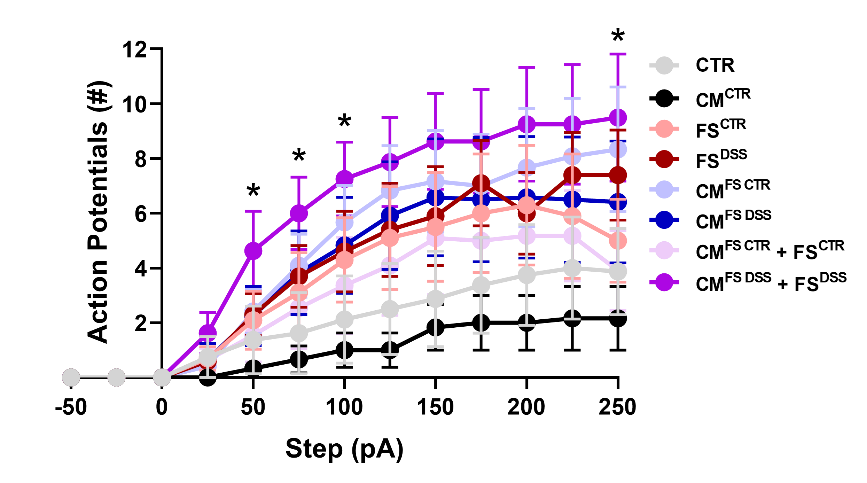
**

**Figure S5. Electrophysiological analysis on DRG neurons exposed to FS and/or CM from colon organoids.**

DRG neurons were exposed for 48 hours to CM^CTR^, FS^CTR^, FS^DSS^, CM^FS CTR^, CM^FS DSS^, CM^FS CTR^ + FS^CTR^ and CM^FS DSS^ + FS^DSS^ and intrinsic excitability was measured. Values represent the mean ± SEM of 6-12 cells analyzed in n=4 experiments. The analysis of variance was performed by One-way ANOVA followed by Bonferroni post hoc comparison. *P<0.05 vs CM^FS CTR^ + FS^CTR^.

**
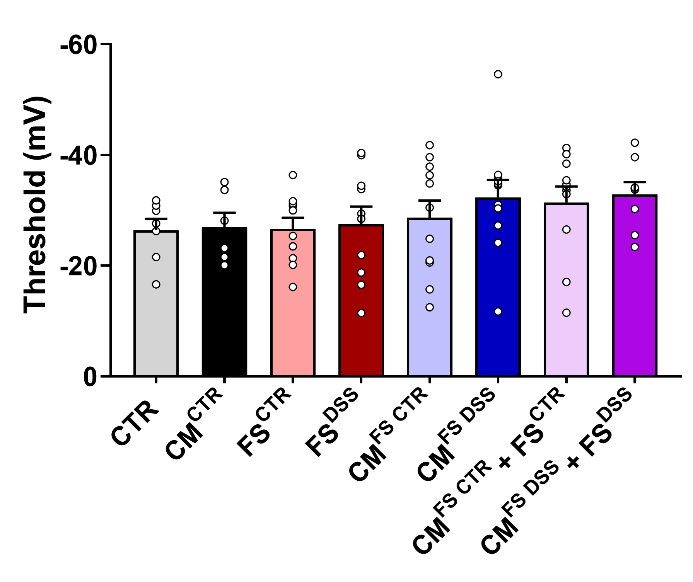
**

**Figure S6. Analysis of the threshold of evoked action potentials of DRG neurons exposed to FS and/or CM from colon organoids.** DRG neurons were exposed for 48 hours to CM^CTR^, FS^CTR^, FS^DSS^, CM^FS CTR^, CM^FS DSS^, CM^FS CTR^ + FS^CTR^ and CM^FS DSS^ + FS^DSS^ and the threshold of evoked action potentials was measured. Values represent the mean ± SEM of 6-12 cells analyzed in n=4 experiments. The analysis of variance was performed by One-way ANOVA followed by Bonferroni post hoc comparison.


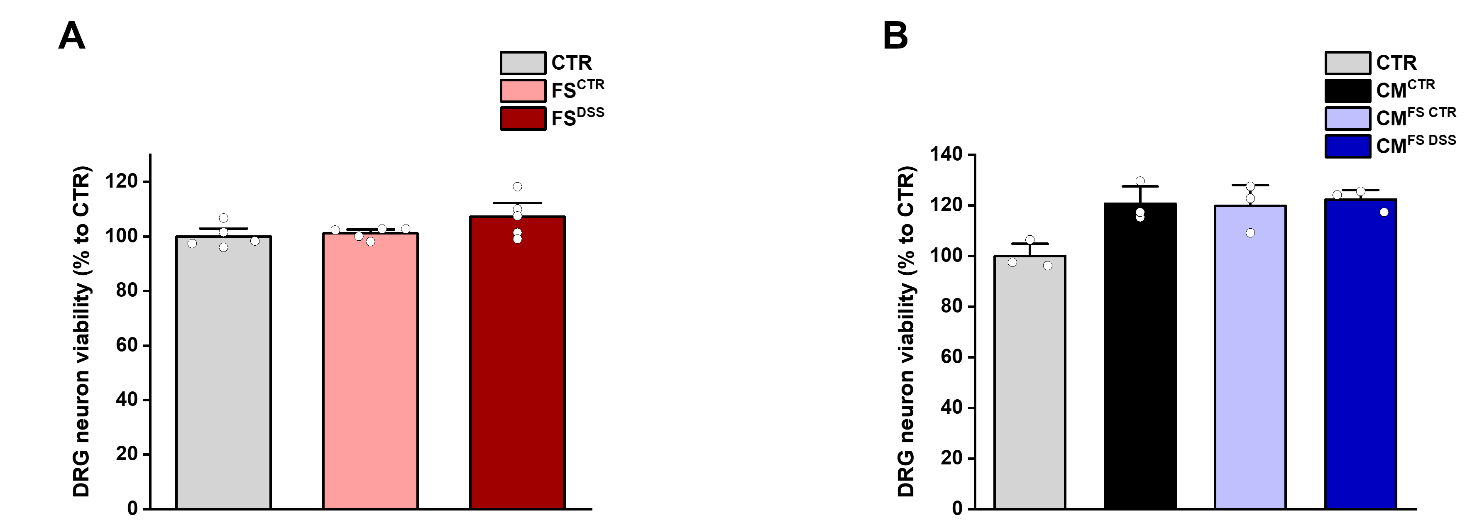


**Figure S7. Effects of FS and CM from organoids on viability of DRG neurons.** Viability was measured on DRG neurons exposed for 48 hours to A) FS^CTR^ and FS^DSS^ and B) CM^CTR^, CM^FS CTR^, CM^FS DSS^. Values represent the mean ± SEM of n=3-5 technical replicates. The analysis of variance was performed by One-way ANOVA followed by Bonferroni post hoc comparison.


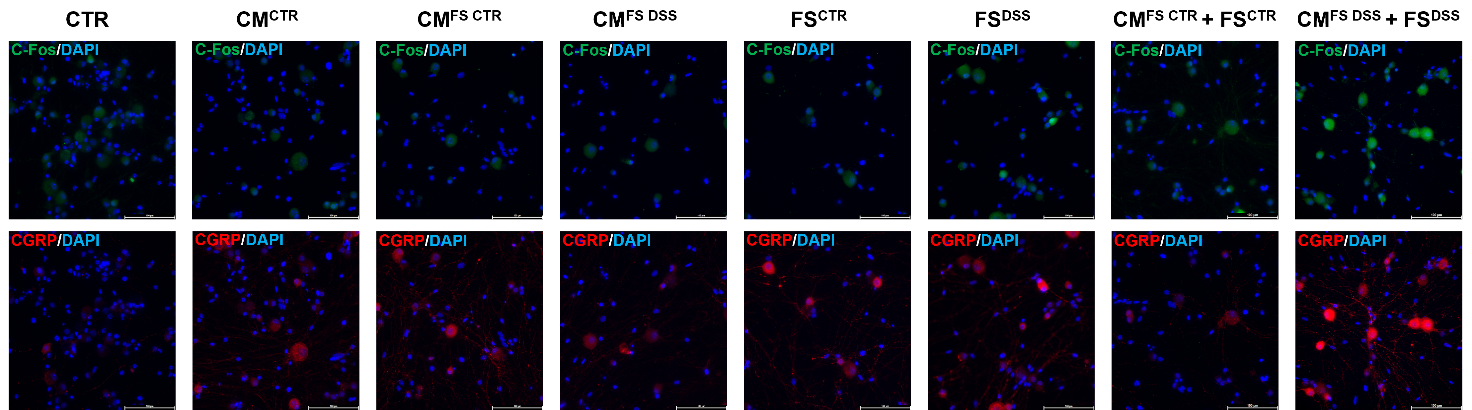


**Figure S8. Immunofluorescence analysis for c-Fos and CGRP in DRG neurons exposed to FS and/or CM from colon organoids.** Representative images of DRG neurons stained for c-Fos (green) and CGRP (red) after 48 hours exposure to CM^CTR^, CM^FS CTR^, CM^FS DSS^, FS^CTR^, FS^DSS^, CM^FS CTR^ + FS^CTR^, and CM^FS DSS^ + FS^DSS^. (Magnification: 40X; scale bar: 100 µm).


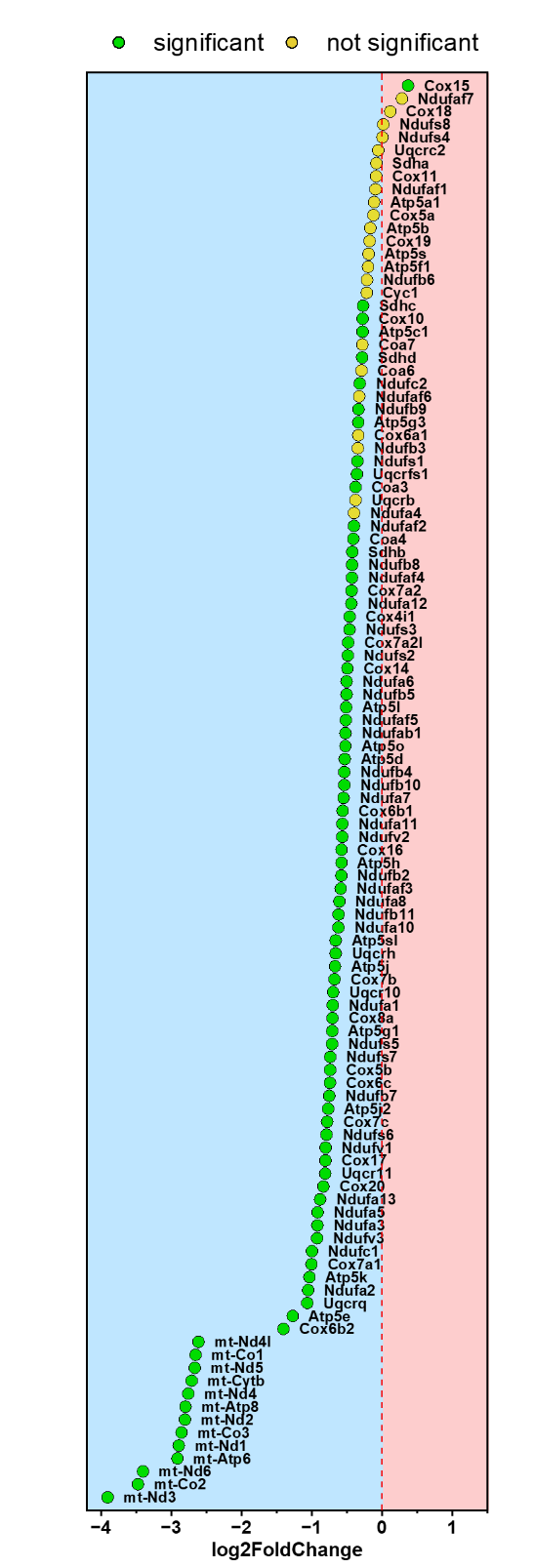


**Figure S9. Expression of genes involved in oxidative phosphorylation in colon organoids under FS^CTR^ and FS^DSS^ treatments.** Genes in the red area were upregulated in FS^DSS^ while genes in the blue area were downregulated in FS^DSS^. Statistical significance was set to P<0.05.


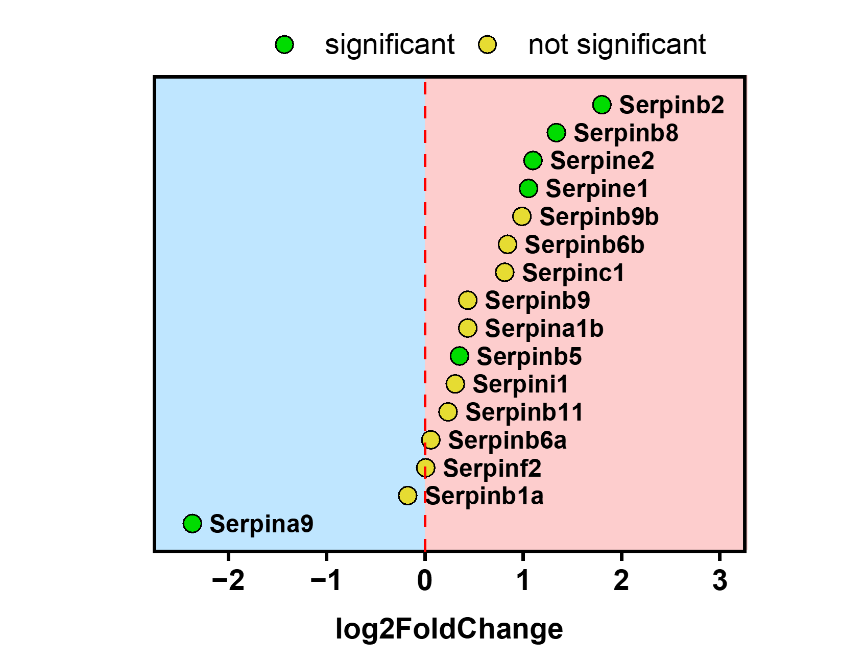


**Figure S10. Expression of genes coding for serpins in colon organoids under FS^CTR^ and FS^DSS^ treatments.** Genes in the red area were upregulated in FS^DSS^ while genes in the blue area were downregulated in FS^DSS^. Statistical significance was set to P<0.05.
